# Disruptions in asymmetric centrosome inheritance and WDR62-Aurora kinase B interactions in primary microcephaly

**DOI:** 10.1038/srep43708

**Published:** 2017-03-08

**Authors:** Paraskevi Sgourdou, Ketu Mishra-Gorur, Ichiko Saotome, Octavian Henagariu, Beyhan Tuysuz, Cynthia Campos, Keiko Ishigame, Krinio Giannikou, Jennifer L. Quon, Nenad Sestan, Ahmet O. Caglayan, Murat Gunel, Angeliki Louvi

**Affiliations:** 1 Departments of Neurosurgery and Neuroscience, Program on Neurogenetics, Yale School of Medicine, New Haven, CT, 06520, USA; 2 Department of Pediatric Genetics, Cerrahpasa Medical School, Istanbul University, Istanbul, Turkey; 3 Departments of Neuroscience, Genetics and Psychiatry, Kavli Institute for Neuroscience, Yale School of Medicine, New Haven, CT, 06520, USA; 4 Department of Medical Genetics, School of Medicine, Istanbul Bilim University, Istanbul, Turkey; 5 Department of Genetics, Yale School of Medicine, New Haven, CT, 06520, USA.

## Abstract

Recessive mutations in *WD repeat domain 62 (WDR62*) cause microcephaly and a wide spectrum of severe brain malformations. Disruption of the mouse ortholog results in microcephaly underlain by reduced proliferation of neocortical progenitors during late neurogenesis, abnormalities in asymmetric centrosome inheritance leading to neuronal migration delays, and altered neuronal differentiation. Spindle pole localization of WDR62 and mitotic progression are defective in patient-derived fibroblasts, which, similar to mouse neocortical progenitors, transiently arrest at prometaphase. Expression of *WDR62* is closely correlated with components of the chromosome passenger complex (CPC), a key regulator of mitosis. Wild type WDR62, but not disease-associated mutant forms, interacts with the CPC core enzyme Aurora kinase B and staining of CPC components at centromeres is altered in patient-derived fibroblasts. Our findings demonstrate critical and diverse functions of WDR62 in neocortical development and provide insight into the mechanisms by which its disruption leads to a plethora of structural abnormalities.

Malformations of cerebral cortical development (MCD) are the outcome of disruptions in the precisely orchestrated series of events that occur during embryonic and early postnatal life and define the specification of neuronal identity and, ultimately, the execution of neuronal differentiation and connectivity both within and outside the cerebral cortex. MCD are neurodevelopmental disorders invariably causing developmental delay and were initially classified according to the phase at which disruptions of normal developmental events were thought to occur, impacting for example neural progenitor proliferation or apoptosis, neuronal migration, or cortical organization^1^,^2^. Progress in our fundamental understanding of normal cortical development^3^,^4^,^5^ along with advances in genetics, genomics, and imaging prompted a revision of the traditional classification^6^. Importantly, early genetic studies led to the realization that a subset of MCD disorders are indeed monogenic (e.g. refs 7, 8, 9, 10, 11), potentially offering unique insights into specific cellular functions involved in brain pathobiology. A wide spectrum of MCD are underlain by genetic lesions^6^, 17 of which are associated with autosomal recessive primary microcephaly (MCPH), a genetically heterogeneous disorder characterized by the clinical finding of a head circumference more than 2 standard deviations below the ethnically-matched age- and sex-related mean and thought to be secondary to abnormal neural progenitor proliferation or apoptosis^6^,^12^,^13^,^14^. MCPH2 (OMIM 604317), the second most common form of primary microcephaly, is caused by recessive mutations in *WDR62*^15^,^16^,^17^, a gene encoding a WD repeat-containing protein. Members of this large pan-eukaryotic protein family are thought to serve as rigid scaffolds coordinating multiprotein complex assemblies with diverse functions, including signal transduction, transcriptional regulation, cilia assembly, cell cycle control and apoptosis^18^,^19^,^20^,^21^. When mutations in *WDR62* were initially discovered^15^, it became apparent that they cause a form of microcephaly invariably accompanied by a wide spectrum of additional and diverse cortical abnormalities, including pachygyria, thickened cortex, lissencephaly, and polymicrogyria, which were traditionally thought to be distinct, suggesting they can have a unified underlying genetic causation. More than 30 missense, nonsense, frameshift or splice site mutations mapping throughout the gene have been reported in patients around the world (refs 15, 16, 17,22, 23, 24, 25, 26, 27, 28, 29, 30, 31, 32 and our unpublished findings). Like many other MCPH-associated proteins, WDR62 has been implicated in spindle maintenance, mitotic progression and maintenance of the neural progenitor pool^33^,^34^,^35^ and further shown to associate with c-Jun N-terminal kinase (JNK) and Aurora kinase A^33^,^34^,^36^,^37^,^38^,^39^. However, the mechanisms by which WDR62 dysfunction results in such diverse spectrum of structural brain abnormalities remain poorly understood.

We investigated WDR62 function using a mouse model and dermal fibroblasts from affected (homozygote) and unaffected (heterozygote) members of a family carrying a novel truncating mutation in *WDR62*. Our studies demonstrated that disruption of WDR62 impairs proliferation of neocortical progenitors during late neurogenesis underlain by abnormalities in asymmetric centrosome inheritance causing microcephaly in mice, and impairs mitotic cycle progression in patient-derived fibroblasts, which, similar to mouse neocortical progenitors, transiently arrest at prometaphase. Moreover, transcriptomic analyses identified genes with expression profiles significantly correlating with *WDR62* across different human brain regions and developmental periods, including those encoding components of the Chromosome Passenger Complex (CPC), a major regulator of mitosis^40^. Functional analyses indicated transient interaction of WDR62 with AURKB, the enzymatic core of the CPC, which is significantly disrupted by MCPH-associated mutant forms of WDR62. Taken together, our findings provide novel mechanistic insight into WDR62 function in cortical development, demonstrating a complex effect on the cell cycle of neocortical progenitors, and may help explain the large spectrum of cortical malformations associated with *WDR62* mutations causing microcephaly.

## Results

### Generation of mutant *Wdr62* ^*1–21*^ mice

*Wdr62* mRNA is expressed in proliferating neural progenitors in the ventricular and subventricular zones (VZ and SVZ, in the latter at higher levels) of the dorsal telencephalon (pallium) as early as E11.5, in ventral (subpallial) progenitors that give rise to striatal neurons and cortical interneurons, and in proliferating granule cell precursors in the external granular layer (EGL) of the cerebellum at early postnatal stages (Supplementary Figure S1)^15^. In adult brain *Wdr62* is highly restricted to the septum and medial thalamus (data not shown). In the developing human brain, *WDR62* mRNA is enriched in the VZ, weakly expressed in the intermediate zone and cortical plate, whereas in adult human brain, it is detected throughout the neocortical layers (Supplementary Figure S1).

To investigate the function of WDR62 we generated a mouse carrying a homozygous mutation in *Wdr62* from trapped ES cells (*Wdr62*^Gt(CH0428)Wtsi^) obtained from the Welcome Trust Sanger Institute (www.informatics.jax.org). The insertion site of the pGTOlxr gene-trap vector is between exons 21 and 22 of the *Wdr62* locus (Supplementary Figure S1). Mice homozygous for the trapped allele express a truncated *Wdr62* transcript as suggested by *in situ* hybridization with a cRNA probe that maps to the 5′ end of *Wdr62*, but not full-length *Wdr62* (Supplementary Figure S1). Mice carrying the trapped allele (henceforth *Wdr62*^*1-21*^) express a fusion protein consisting of amino acids 1–870 (of 1524) of WDR62 fused to the β-geo reporter, detected by x-gal staining, in accordance to the endogenous pattern (Supplementary Figure S1). *Wdr62*^*1-21*^homozygous mice are viable but infertile, exhibiting dramatically reduced pregnancy rates (only two litters were produced in a period of six months by seven breeding pairs with one of the breeders being homozygous) and offspring number, likely reflecting defects in the germline. Several MCPH2- associated mutations identified in patients also lead to C-terminal truncations of variable length (e.g. S956CfsX38^16^, G1280AfsX21^15^,^16^, V1314RfsX18/V1313GfsX17^16^,^17^,^25^, V1402GfsX12^15^ and L1414LfsX41^17^) and a novel allele reported herein (D955AfsX112; see below).

### Disruption of *Wdr62* causes microcephaly in mice

**Figure 1:**
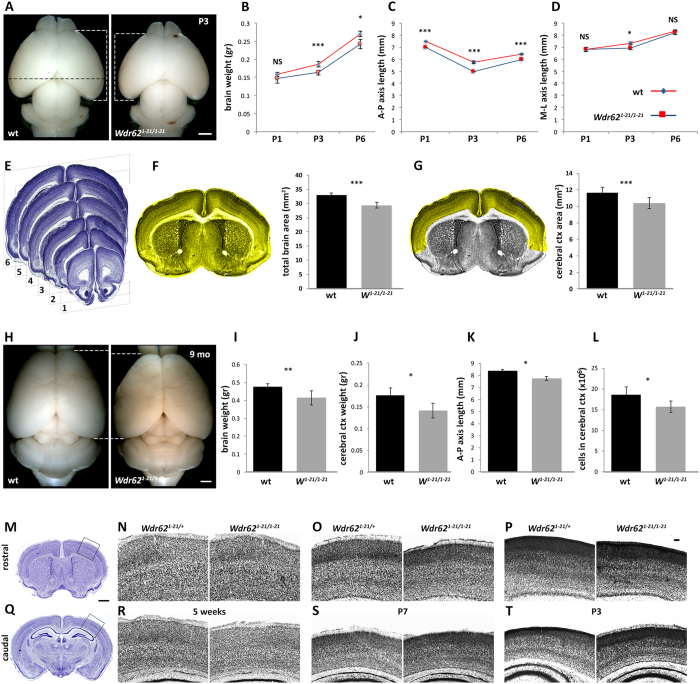
Microcephaly and abnormal brain cytoarchitecture in *Wdr62*^*1-21/1-21*^ mice. (**A–L**) *Wdr62*^*1-21/1-21*^mice exhibit microcephaly. (**A**) Representative images of whole brains from WT and *Wdr62*^*1-21/1-21*^ littermates at P3. (**B**) Brain weight is significantly decreased in *Wdr62*^*1-21/1-21*^ pups compared with WT at P3 and P6. (**C,D**) Measurements of the anterior to posterior (**A–P**) white dotted brackets in (**A**) and medial to lateral (M-L, black dotted line in (**A**) axis length of WT and *Wdr62*^*1-21/1-21*^ forebrains at P1, P3 and P6 show significant differences in (**A–P**) axis length (**C**) and a transient decrease in (M-L axis length at P3 (**D**) in *Wdr62*^*1-21/1-21*^ forebrains compared with WT. Measurements were obtained from at least 3 and up to 9 pairs of brains per stage. (**E–G**) Total brain and cerebral cortical area are reduced in *Wdr62*^*1-21/1-21*^mice. (**E**) Measurements were performed at six coronal levels along the A-P axis as indicated (n = 6 pairs, 6 sections per brain analyzed). (**F,G**) Significantly smaller brain (highlighted in F) and cerebral cortical (highlighted in G) areas in *Wdr62*^*1-21/1-21*^ animals compared with WT. (**H**) Representative images of whole brains from WT and *Wdr62*^*1-21/1-21*^ littermates at 9 weeks of age. (**I–K**) Reduced weight of whole brain (**I**) and cerebral cortex (**J**) and reduced length of A-P (**K**), but not M-L (see Figure S1), axis in *Wdr62*^*1-21/1-21*^ mice compared with WT. (**L**) Decrease in total cell number in cerebral cortex and hippocampus of *Wdr62*^*1-21/1-21*^ mice compared with WT as determined by the isotropic fractionator method (n = 4 pairs). (**M–T**) *Wdr62*^*1-21/1-21*^mice exhibit abnormal cytoarchitecture in the cerebral cortex. Analyses of Nissl stained coronal brain sections at rostral (**M–P**) and caudal (**Q–T**) A-P levels at 5 weeks (**M,N,Q,R**), P7 (**O,S**) and P3 (**P,T**) reveals reduced thickness and abnormalities in neocortical cytoarchitecture in *Wdr62*^*1-21/1-21*^brains compared with control (as indicated). At early postnatal stages, upper cortical layers appear condensed and deep layers are disorganized. These defects are more pronounced rostrally. In panels N–P and R–T, sections are shown in a medial (to the left) to lateral (to the right) orientation. Error bars represent s.e.m; ^★^p < 0.05, ^★★^p < 0.01, ^★★★^p < 0.005; NS: not significant (two-tailed Student’s *t*-test) Scale bar: (**A,H**): 1 mm; (**M,Q**): 0.5 mm; (P; applies to **N–P** and **R–T**): 0.1 mm.

*Wdr62*^*1-21/1-21*^mice were smaller than wild type at early postnatal stages, but no longer in adulthood (Supplementary Figure S2); however, their brain was smaller than normal from birth onwards (Fig. 1A,H and Supplementary Figure S2). They showed no spontaneous pathologies during a four-year period of observation. Measurements of cortical dimensions in the first postnatal week showed significant reduction in the length of the anterior to posterior (A-P) axis (wild type vs. *Wdr62*^*1-21/1-21*^: P1: 7.5 mm ± 0 vs. 7 mm ± 0; P3: 5.75 mm ± 0.111 vs. 5 mm ± 0; p = 0.001, P6: 6.44 mm ± 0.055 vs. 6mm ± 0.083; p = 4.36 × 10^−5^) but no difference in the medial to lateral axis (M-L, measured at the widest part of the forebrain), with the exception of P3 brains (n = 3–9 pairs of wild type and *Wdr62*^*1-21/1-21*^brains [P1: 6.83 mm ± 0.166 vs. 6.83 mm ± 0.166; P3: 7.33 mm ± 0.105 vs. 6.91 mm ± 0.083; p = 0.04, P6: 8.33 mm ± 0.083 vs. 8.22 mm ± 0.168; p = 0.512]) (Fig. 1B–D, and Supplementary Figure S2). In addition, whole brain and neocortical areas were significantly reduced (wild type vs. *Wdr62*^*1-21/1-21*^: 32.888 mm^2^ ± 0.887 vs. 29.390 mm^2^ ± 0.991; p = 2.56 × 10^−6^ and 11.638 mm^2^ ± 0.637 vs. 10.376 mm^2^ ± 0.637; p = 1.608 × 10^−4^ respectively), measured at different levels along the rostral to caudal axis at P3 (n = 6 pairs, with six sections analyzed per brain) (Fig. 1E–G). In adulthood, the average brain weight (wild type vs. *Wdr62*^*1-21/1-21*^: 0.476 gr ± 0.008 vs. 0.415 gr ± 0.02; p = 0.006, n = 5) or of cerebral cortical (0.176 gr ± 0.029 vs. 0.141 gr ± 0.035; p = 0.029, n = 3) weight was significantly reduced, as was the length of the A-P axis (8.375 mm ± 0.125 vs. 7.75 mm ± 0.144; p = 0.016, n = 4) (Fig. 1H–K). By applying the isotropic fractionator method^41^ we determined that total cell number in the neocortex and hippocampus (9 weeks of age, (n = 4 pairs of brains from littermates) was significantly reduced in *Wdr62*^*1-21/1-21*^(15,731,250 ± 1,369,815) compared with wild type (18,559,375 ± 1,874,669, p = 0.01) (Fig. 1L). Nuclear volume was similar in neocortical neurons from wild type and *Wdr62*^*1-21/1-21*^P3 brains immunostained with an antibody against BCL11B (aka CTIP2; a transcription factor enriched in subcerebrally projecting neurons in layers 5 and 6) (Supplementary Figure S2), suggesting that reduced cell number was the likely reason for smaller brain size in *Wdr62*^*1-21/1-21*^mice. Cortical radial thickness was also reduced and neocortical cytoarchitecture was disrupted (Fig. 1M–T), with dense and compact upper cortical layers, especially at rostral levels, without, however, perturbed cell distribution (Supplementary Figure S2). These differences appeared to be transient but, although clearly evident and reproducible in the first postnatal week (Fig. 1O,P,S,T), were less pronounced in young adult brain (Fig. 1N,R). At late embryonic and early postnatal stages (P1, P3) the lateral ventricles of *Wdr62*^*1-21/1-21*^brains appeared enlarged (Supplementary Figure S2). These observations demonstrated that WDR62 is a critical regulator of neocortical size in mice, as in humans, suggesting evolutionarily conserved function.

### WDR62 is required for proliferation of late-born cortical progenitors

**Figure 2:**
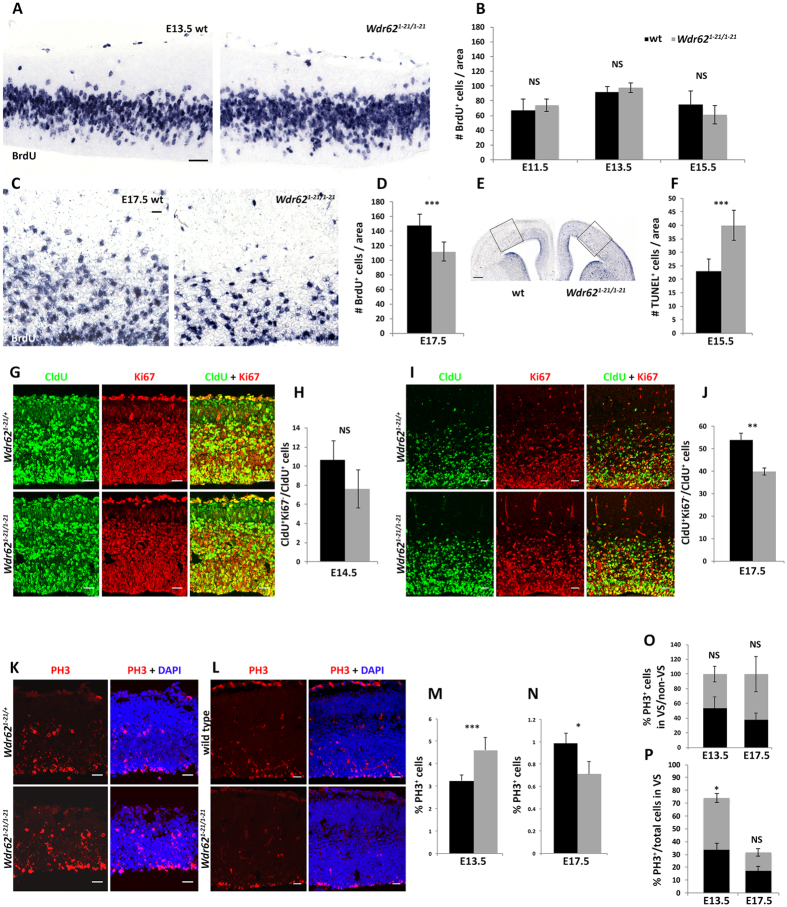
Proliferation and cell cycle defects in *Wdr62*^*1-21/1-21*^ neural progenitors. (**A–D**) BrdU incorporation analyses. (**A,C**) Coronal sections of wild type and *Wdr62*^*1-21/1-21*^ embryonic brains at E13.5 (**A**) and E17.5 (**C**) immunostained with an antibody specific to BrdU. Embryonic neocortices were analyzed following a 30-minute BrdU pulse. (**B,D**) Quantification of the number of BrdU^+^ cells at E11.5, E13.5, E15.5 (**B**) and E17.5 (**D**) reveals decreased proliferation in *Wdr62*^*1-21/1-21*^ brains compared with wild type at late stages of neocortical development (n = 3 pairs, 3–4 sections analyzed per brain). (**E,F**) Detection of apoptotic cells using the TUNEL assay. Quantification of cells stained with TUNEL in coronal brain sections (**E**) reveals increased apoptosis (**F**) at E15.5 in *Wdr62*^*1-21/1-21*^ neocortex compared with wild type (n = 3 pairs of brains, 3 sections analyzed per brain; boxes in E delineate the area of each section where cells were counted). (**G**–**J**) Analyses of cell cycle exit. Immunofluorescence staining for CldU and Ki67 at E14.5 (**G**) and E17.5 (**I**) following a 24-hour CldU pulse. (**H,J**) Quantification of CldU^+^ Ki67^−^/CldU^+^ cells indicates reduced cell cycle exit at late stages of neurogenesis (E17.5) in *Wdr62*^*1-21/1-21*^ compared with wild type brains (n = 3–4 pairs of brains, 3–4 sections analyzed per brain). (**K–P**) Analyses of progenitor cell division. (**K,L**) Immunofluorescence staining for PH3 at E13.5 (**K**) and E17.5 (**L**). (**M–P**) Quantification of PH3^+^ cells reveals significantly more mitotic cells in the developing neocortex of *Wdr62*^*1-21/1-21*^ embryos compared with wild type littermates at E13.5 (**M**). In contrast, at the end of neurogenesis, the number of mitotic cells is decreased in *Wdr62*^*1-21/1-21*^ neocortex (**N**). There are no significant differences in the distribution of PH3^+^ cells at the ventricular surface (VS) or at a distance from it between wild type and *Wdr62*^*1-21/1-21*^ embryonic neocortex (**O**). Mitotic index (PH3^+^ cells/total number of cells) in the VS is increased in *Wdr62*^*1-21/1-21*^ neocortex compared with wild type at E13.5, but not significantly different at E17.5 (**P**) (n = 3–4 pairs, 3–4 sections analyzed per brain). Number of DAPI^+^ cells counted per section in wild type vs. *Wdr62*^*1-21/1-21*^: 1,075 vs. 934 (E13.5); 3,059 vs. 3,350 (E17.5). Error bars represent s.e.m; ^★^p < 0.05, ^★★^p < 0.01, ^★★★^p < 0.005; NS: not significant (two-tailed Student’s *t*-test) Scale bar: (**A,C**) 20 μm; (**G,I,K,L**) 30 μm.

A small brain with fewer cells may result from premature depletion of the neural progenitor pool and/or increased apoptosis. We first analyzed BrdU incorporation throughout embryogenesis, failing to detect significant differences in cortical progenitor cell proliferation in wild type vs. *Wdr62*^*1-21/1-21*^ brain at E11.5, E13.5 and E15.5 following a 30-minute pulse (Fig. 2A,B). At E17.5, however, progenitor proliferation was reduced (Fig. 2C,D). TUNEL assay demonstrated more apoptotic cells in dorsal and ventral telencephalon of *Wdr62*^*1-21/1-21*^ embryos compared with wild type at E15.5 (Fig. 2E,F), suggesting that the reduction in radial cortical thickness may be partially due to increased apoptosis in mid- and late neurogenesis. To assess cell cycle dynamics in dividing neural progenitors, we injected CldU at early (E13.5) and late (E16.5) stages of neurogenesis, and harvested the brains 24 hours later. We quantified the percentage of cells that had exited the cell cycle (CldU^+^Ki67^−^/CldU^+^) during the 24-hour chase period by double labeling with antibodies specific for CldU and Ki67, which labels all dividing cells. The 24-hour cell cycle exit index was not significantly different in embryos injected at E13.5 and harvested at E14.5 (Fig. 2G,H), but was reduced in *Wdr62*^*1-21/1-21*^embryos injected at E16.5 and harvested at E17.5 compared with wild type (Fig. 2I,J). Additionally, the “leaving fraction” (BrdU^+^Ki67^−^)^42^, comprising cells in S phase at the time of BrdU injection that exited the cell cycle during the 30-minute chase period, was increased in *Wdr62*^*1-21/1-21*^vs. wild type neocortex at E17.5 (Supplementary Figure S3). Quantification of mitotic cells (expressing phosphor-histone H3 [PH3]) at early (E13.5) and late (E17.5) stages of neurogenesis revealed that the mitotic index was increased in *Wdr62*^*1-21/1-21*^embryos at E13.5, but decreased at E17.5 (Fig. 2K–N). The distribution of progenitors dividing in the ventricular surface (VS) or away from it was similar in the two genotypes at both stages examined (Fig. 2O). At the VS, we counted considerably more PH3^+^ cells in *Wdr62*^*1-21/1-21*^embryonic brains at E13.5 compared with wild type (Fig. 2P). Therefore, WDR62 disruption appeared to impact early and late neural progenitors differently: at early stages, it resulted in increase in the number of progenitors undergoing mitosis without, however, significantly affecting proliferation or cell cycle exit, whereas by late neurogenesis, WDR62 disruption led to decreased proliferation and decreased 24-hour cell cycle exit index, yet increased “leaving fraction” over a 30-minute period, suggesting that some (but not all) late progenitors leave the cell cycle early. Taken together these observations suggest that WDR62 differentially impacts self-renewal and differentiation of early vs. late neocortical progenitors.

### WDR62 is required for proper centrosome inheritance

**Figure 3:**
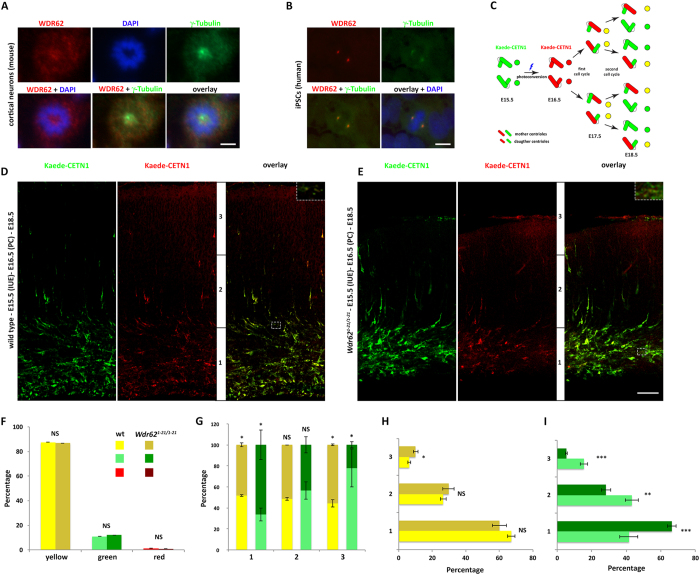
Defective centrosome inheritance in *Wdr62*^*1-21/1-21*^ neocortex. (**A,B**) Immunofluorescence staining of murine neocortical progenitors (**A**) (prometaphase) and human induced pluripotent stem cells (iPSCs) (**B**) (metaphase) with antibodies specific to WDR62 and γ-tubulin. (**C**) Experimental procedure to visualize centrosomes with mother centrioles of different ages^44^. Centrosomes replicate once per cell cycle, giving rise to two centrosomes, each consisting of one mother and one daughter centriole transiently labeled via in utero electroporation with Kaede-CETN1 that is photoconvertable from green to red fluorescence. After the first replication, new centrosomes harbor one mother (red) and one daughter (green) centriole. After the second -and subsequent- cycles, centrosomes retaining the mother (red) and a newly synthesized (green) centriole appear yellow whereas those inheriting the daughter (green) and a newly synthesized centriole are green-fluorescent. (**D,E**) Representative images of the cortical wall of E18.5 WT (**D**) and *Wdr62*^*1-21/1-21*^ (**E**) littermates electroporated with Keade-CETN1 at E15.5 and photoconverted at E16.5. Insets are digital magnification images of the areas delineated by dotted squares depicting centrosomes that are green-only, red-only, or yellow-fluorescent. Some cells show protein accumulation in the cytoplasm as well (See also Supplementary Figure 4). The cortical wall was divided into three equally sized bins (1: VZ/SVZ; 2: IZ; 3: cortical plate). (**F,G**) Quantification of the percentage of green-, yellow-, or red- (**F**) and of green- or yellow-fluorescent centrosomes, as a percentage of all centrosomes, within each bin (**G**). *Wdr62*^*1-21/1-21*^ brains harbor significantly more green centrosomes in bin 1 and fewer in bin 3, and fewer yellow centrosomes in bin 1 and more in bin 3. (**H,I**) Distribution of green- and yellow centrosomes. *Wdr62*^*1-21/1-21*^ brains harbor a higher percentage of yellow centrosomes in bin 3 (**H**). In bin 1, and in bins 2 and 3, they also harbor, respectively, a higher and a lower percentage of green centrosomes (**I**). (number of centromeres counted: 7,156 [WT] vs. 6,960 [*Wdr62*^*1-21/1-21*^] from 6 sections per brain from three pairs of brains). Error bars represent s.e.m; ^★^p < 0.05, ^★★^p < 0.01, ^★★★^p < 0.005; NS: not significant (two-tailed Student’s *t*-test). Scale bar: (**A**) 12.5 μm; (**B**) 20 μm; (**E**) also applies to (**D**) 50 μm.

In non-neuronal cells, and dependent on cell line examined, WDR62 is detected at the spindle poles during at least some phases of mitosis^15^,^17^,^22^,^27^,^33^,^35^,^43^ and appears to associate with the centrosome, although it is not principally a centrosomal protein, at least in HeLa cells^33^. We re-examined WDR62 expression in neural progenitors, as we previously could not detect clear localization of WDR62 to the spindle poles *in vivo* using antibodies available at the time^15^. WDR62 can be detected at the spindle poles of cultured mouse neocortical progenitors, dependent on experimental conditions (Fig. 3A) and in human induced pluripotent stem cells (iPSCs) (Fig. 3B). This association with the spindle poles, together with the effects of WDR62 dysfunction on mitotic index and progenitor proliferation (Fig. 2), prompted us to investigate asymmetric centrosome inheritance as a possible mechanism underlying proliferation defects^44^. We utilized an *in vivo* assay (developed by S.-H. Shi^44^) to pulse-label centrosomes of proliferating neocortical progenitors with the photoconvertible fluorescent protein Kaede^45^ fused to the centriolar protein centrin 1 (Kaede-CETN1, gift from S-H Shi), in order to distinguish centrosomes containing the old mother centriole from those containing the new mother centriole (Fig. 3C). Kaede-CETN1 was electroporated at E15.5 and photoconverted at E16.5 (Fig. 3D,E and Supplementary Figure S4). In wild type as well as *Wdr62*^*1-21/1-21*^neocortex at E18.5, more than 85% of labeled centrosomes contained both green and red (“yellow” upon merge) fluorescent centrioles; about 10% of centrosomes possessed only green fluorescent centrioles, whereas less than 5% were solely red fluorescent (corresponding to initially labeled progenitors that had not divided during this period), in agreement with previous observations^44^ (Fig. 3F). However, within the proliferating zones (VZ/SVZ; bin 1), the percentage of centrosomes with only green fluorescent centrioles (i.e. containing new mother centrioles only, having undergone two rounds of cell division following photoconversion) was higher in *Wdr62*^*1-21/1-21*^compared with wild type (65% vs. 40%), whereas, the percentage of those retaining the old mother centriole (both green and red fluorescent, i.e. “yellow” upon merge) was lower (Fig. 3G–I). The opposite was true for the cortical plate (bin 3): compared with wild type, the *Wdr62*^*1-21/1-21*^brains had fewer centrosomes containing green only centrioles (Fig. 3G–I) and a higher percentage of centrosomes containing both green and red centrioles (i.e. “yellow” upon merge) (Fig. 3G–I). These data suggested that, compared with wild type, in the proliferating zone of *Wdr62*^*1-21/1-21*^neocortex more cells inherited the new mother centriole (green), which is typically inherited by the newly differentiating neurons migrating away from the VZ/SVZ. Accordingly, the distribution of cells containing new mother centrioles only (i.e. green fluorescent) within the developing neocortex (bin 1, 2 and 3), was different in *Wdr62*^*1-21/1-21*^compared with wild type neocortex, with the former harboring significantly more in the proliferating zones and fewer in the migration zone and the cortical plate, suggesting abnormal migration, and possibly differentiation, of *Wdr62*^*1-21/1-21*^neurons inheriting the new mother centriole.

### Abnormalities in neuronal differentiation in *Wdr62* ^*1-21/1-21*^mice

**Figure 4:**
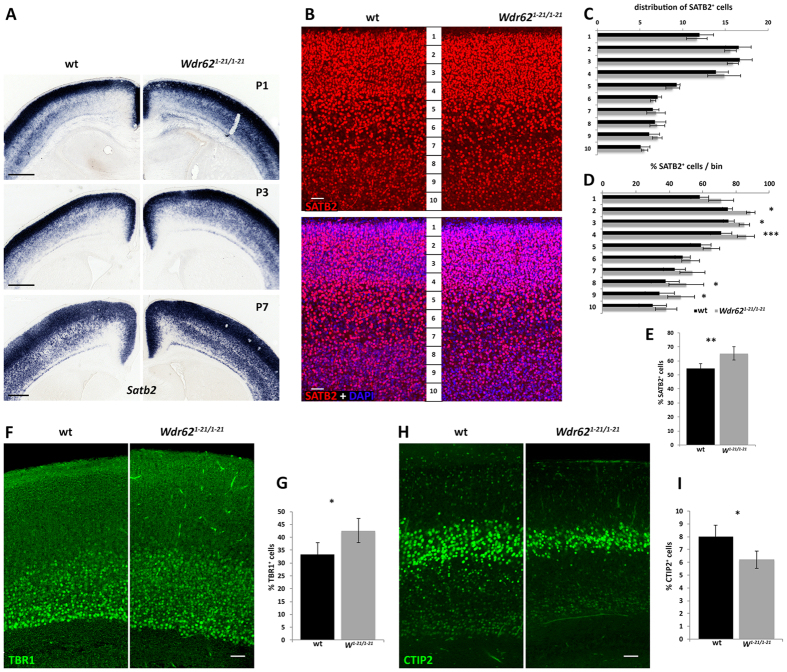
Abnormalities in neuronal differentiation in *Wdr62*^*1-21/1-21*^ brains. (**A–I**) Neuronal differentiation defects in *Wdr62*^*1-21/1-21*^ brains revealed by *in situ* hybridization and immunofluorescence staining with markers of post-mitotic cortical neurons. (**A**) Representative images of coronal brain sections from wild type and *Wdr62*^*1-21/1-21*^ brains processed with *in situ* hybridization for *Satb2* at the stages indicated. *Wdr62*^*1-21/1-21*^ brains show an expansion of *Satb2* mRNA expression domain compared with wild type. (**B–E**) Representative immunofluorescence images of coronal sections from wild type and *Wdr62*^*1-21/1-21*^ brains at P3 stained with an antibody specific to SATB2 (**B**). Quantification of SATB2^+^ cells within each of 10 equally sized bins in relation to the total number of SATB2^+^ cells demonstrates normal distribution (**C**). Quantification of cortical neurons that are SATB2^+^, in relation to the total number of cortical cells (DAPI^+^) within each bin, demonstrates an increase in number of SATB2^+^ cells in upper (bins 2–4) and deep layers (bins 8 and 9, mostly layer 6) (**D**). The overall fraction of SATB2^+^ neurons is increased in *Wdr62*^*1-21/1-21*^ brains compared with wild type (n = 4 pairs, average number of DAPI^+^ cells counted per section: 5,469 [wild type] vs. 5,320 [*Wdr62*^*1-21/1-21*^]) (**E**). (**F–I**) Representative immunofluorescence images of coronal sections from wild type and *Wdr62*^*1-21/1-21*^ brains at P3 stained with antibodies specific to TBR1 (**F**) or CTIP2 (**H**). Quantification of the fraction of TBR1^+^ or CTIP2^+^ neurons, in relation to the total number of cortical cells (DAPI^+^), shows that the percentage of TBR1+ cells is increased in *Wdr62*^*1-21/1-21*^ brains compared with wild type (n = 3 pairs, average number of DAPI^+^ cells counted per section: 3,341 [wild type] and 3,207 [*Wdr62*^*1-21/1-21*^]) (**G**), whereas the percentage of CTIP2^+^ cells is decreased (**I**) (n = 4 pairs, average number of DAPI+ cells counted per section: 3,518 [wild type] vs. 3,504 [*Wdr62*^*1-21/1-21*^]). Error bars represent s.e.m; ^★^p < 0.05, ^★^p < 0.01, ^★★★^p < 0.005; NS: not significant (two-tailed Student’s *t*-test). Scale bar: (**A**) shown on each panel): 0.5 mm; (**B,E,G**) 50 μm.

Disruption in proliferation and cell cycle progression of progenitors has been linked to perturbations in neuronal cell fate^46^,^47^,^48^,^49^,^50^. At mid-to-late embryonic stages, mRNA expression of molecular markers of neural progenitors or subsets of post-mitotic neurons was grossly normal in *Wdr62*^*1-21/1-21*^ neocortex (Supplementary Figure S5). At early postnatal stages, however, expression of *Satb2*, a marker of callosal projection neurons^51^,^52^ appeared increased in *Wdr62*^*1-21/1-21*^neocortex compared with wild type (Fig. 4A). We quantified SATB2-expressing neurons after dividing the neocortical wall into 10 equally sized bins (Fig. 4B). Although their distribution (SATB2^+^ cells in each bin over all SATB2^+^ cells) was similar in wild type and *Wdr62*^*1-21/1-21*^neocortex (Fig. 4C), more cells expressed SATB2 in the latter both in deep (bins 8 and 9) but also more superficial (bins 2–4) parts (Fig. 4D), leading to an overall increase in the percentage of SATB2^+^ neurons (Fig. 4E). In agreement with this, the corpus callosum appeared thicker in *Wdr62*^*1-21/1-21*^brains compared with wild type (Supplementary Figure S6). Furthermore, more neurons expressed TBR1 (a marker for most pyramidal neurons in layers 1–3 and 6^53^) and, conversely, fewer expressed CTIP2 (subcerebrally projecting neurons;^54^) in *Wdr62*^*1-21/1-21*^neocortex compared with wild type (Fig. 4F–I). At P21, and in adult, several cortical layer markers appeared normal (Supplementary Figure S6 and data not shown), suggesting that despite abnormal cytoarchitecture in the neonatal period, cortical lamination was grossly normal in adult *Wdr62*^*1-21/1-21*^ mice. This is in contrast to what is observed in patients with WDR62-associated MCPH, who invariably present with diverse malformations of cortical development (e.g. refs 15, 16, 17). Taken together, these data suggested that loss of WDR62 in neural progenitors affects the differentiation of a subset of cortical neurons.

### Whole-exome sequencing identifies a novel recessive mutation in *WDR62* in patients with microcephaly and structural brain abnormalities

**Figure 5:**
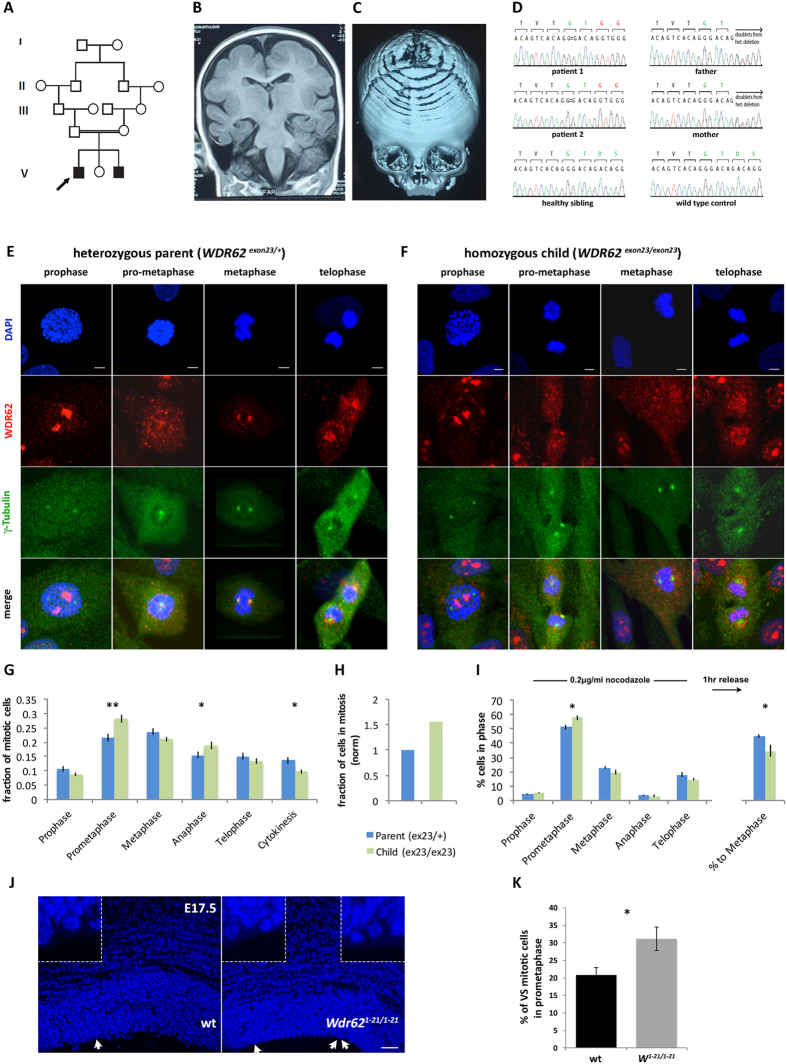
WDR62 is essential for mitotic cycle progression in human fibroblasts and mouse embryonic neocortical progenitors. (**A–D**) Characterization of kindred NG1406 with *WDR62*-associated microcephaly. (**A**) Pedigree structure depicting a second-cousin consanguineous union. Arrow indicates the index case. (**B,C**) Coronal T1-weighted images demonstrate pachygyria (**B**) and 3D reconstruction of computed tomography scanned images show metopic synostosis (**C**) in the index case. (**D**) Chromatograms obtained via Sanger sequencing analysis of the two affected patients, their healthy sibling, and their parents, along with wild type control DNA (as indicated). Sanger sequencing confirmed exome sequencing findings of a 4-bp deletion in exon 23 of *WDR62* resulting in a frameshift (D955AfsX112; designated *WDR62*^*exon23*^). The mutation was confirmed as being homozygous in the two affected siblings and heterozygous in their parents and was absent from the healthy sibling. (**E–I**) Analyses of fibroblasts from family NG1406. (**E,F**) Fibroblast cultures established from the heterozygous parents and the two affected siblings immunostained with antibodies specific to WDR62 and γ-tubulin demonstrate dynamic expression of WDR62 during the mitotic cycle. In heterozygous cells (**E**), WDR62 is detected at the spindle poles in prometaphase through telophase; in *WDR62*^*exon23*^homozygous cells (**F**), WDR62 remains diffusely distributed. (**G–I**) Distribution of mitotic cells in different phases of the mitotic cycle in synchronized cultures. Increased fraction of mitotic cells in prometaphase and anaphase, and decreased fraction of mitotic cells in cytokinesis are observed in *WDR62*^*exon23*^homozygous compared with heterozygous cultures (**G**). The fraction of cells undergoing mitosis is increased in homozygous cultures (**H**). Fewer cells proceed to metaphase following nocodazole-mediated arrest at prometaphase and subsequent release in normal culture conditions in *WDR62*^*exon23*^homozygous as compared with heterozygous cultures (**I**). (**J,K**) Quantification of the distribution of cells in each phase of the mitotic cycle along the ventricular surface (VS) of the developing neocortex (stained with DAPI; J) reveals more mitotic cells in prometaphase in *Wdr62*^*1-21/1-21*^embryos compared with wild type (**K**). Arrows in (**J**) indicate mitotic cells along the VS, which are digitally magnified in the corresponding insets. Error bars represent s.e.m; ^★^p < 0.05, ^★★^p < 0.01, ^★★★^p < 0.005 (two-tailed Student’s *t*-test). Scale bar: (**E,F**) 5 μm; (**J**) 30 μm.

We previously reported six independent MCPH-associated mutations in *WDR62*^15^ and many more have been described since by others. We identified a family (NG1406) with two affected siblings displaying global developmental delay (Fig. 5A and Supplementary data). The index case is a 13-year-old full-term male, the product of a consanguineous (second-cousin) marriage. He presented for medical attention at 20 months of age owning to global developmental delay and was found on clinical examination to have microcephaly and dysmorphic face. Neuroimaging studies identified an array of developmental abnormalities including diffuse pachygyria, thickened cortex and hypoplasia of the corpus callosum. 3D computed tomography showed metopic synostosis (Fig. 5B,C). His 6-year-old brother displayed similar findings (Supplementary data). Whole-exome sequencing identified a novel frameshift mutation in *WDR62*, D955AfsX112 (henceforth *WDR62*^*exon23*^), caused by a 4 bp deletion in exon 23, resulting in a premature stop codon at position 1067; the mutation was confirmed to be homozygous in both affected subjects, and heterozygous in both parents, using Sanger sequencing (Fig. 5D). It was not observed in more than 1,800 Turkish control chromosomes, the ESP (exome sequencing project) or 1,000 genome databases, and was detected twice in heterozygous state in the ExAC database with 1.649E-05 allele frequency. The *WDR62*^exon23^ mutation results in a truncated protein that lacks 569 C-terminal amino acids.

### Mutations in *WDR62* disrupt mitotic cycle progression

We characterized WDR62 expression in primary fibroblasts obtained from skin biopsies from the NG1406 family above carrying the *WDR62*^*exon23*^ allele. In cells from the heterozygous unaffected parents, WDR62 was diffusely distributed in prophase, progressively localizing to the spindle and spindle poles at prometaphase through late metaphase/anaphase, and dispersing again during telophase and cytokinesis (Fig. 5E). In contrast, in cells from the homozygous affected siblings, WDR62 did not localize to the spindle poles (Fig. 5F). Mitotic spindle formation appeared normal in fibroblasts of either genotype, as well as in iPSCs reprogrammed from them (Supplementary Figure S7); very rarely, extra centrosomes were observed (Supplementary Figure S8F; low frequency precluded statistical analyses). The integrity of the spindle poles in homozygous fibroblasts was further demonstrated by normal localization of ASPM, whose loss of function causes primary microcephaly (MCPH5)^55^,^56^, as well as of CDK5RAP2, a pericentriolar protein linked to MCPH3^57^,^58^ (Supplementary Figure S8). Moreover, the vast majority of centrosomes formed normally in *WDR62*^*exon23*^heterozygous and homozygous fibroblasts, as evidenced by normal expression of CEP135 (Figure S8), which also causes MCPH when mutated^59^,^60^. Recently, WDR62 was reported to shuttle CEP63 to the centrosome^43^, however, in *WDR62*^*exon23*^homozygous fibroblasts, CEP63 localization was not disrupted (Supplementary Figure S8). Finally, we observed an increase in red-fluorescent centrosomes in homozygous as compared with heterozygous fibroblasts following transfection with Kaede-CETN1 and photoconversion (Supplementary Figure S8) suggesting that WDR62 disruption delays cell cycle progression.

In synchronized cultures, we observed a higher frequency of *WDR62*^*exon23*^ homozygous fibroblasts in prometaphase and anaphase (Fig. 5G). This increase was only partly counterbalanced by a decrease in cells in cytokinesis (Fig. 5G), such that the fraction of cells in mitosis was significantly higher in *WDR62*^*exo23*^ homozygous cultures (Fig. 5H). Thus, after an initial delay, *WDR62*^*exon23*^ homozygous cells were able to complete mitosis. We examined the capacity of *WDR62*^*exon23*^fibroblasts to proceed into the mitotic cycle following nocodazole-mediated arrest in prometaphase and subsequent release into normal culture media for one hour and observed a delay in homozygous compared with heterozygous cultures as reflected in lower frequency of metaphase cells in the former (Fig. 5I). In agreement with these findings, we observed significantly more mitotic progenitors at prometaphase in the VS of E17.5 *Wdr62*^*1-21/1-21*^neocortex compared with wild type (Fig. 5J,K). Taken together, these observations suggested that WDR62 disruption is associated with abnormalities in mitotic cell cycle progression in patient fibroblasts as well as in embryonic murine neocortical progenitors.

To evaluate the functional consequences of additional *WDR62* mutations at the cellular level, we used HeLa cells, which express WDR62 at higher levels throughout mitosis (as early as prophase and until after telophase) compared with other cells, including fibroblasts (Supplementary Figure S9). These analyses revealed a previously unappreciated dynamic expression during mitosis, with WDR62 initially diffusely distributed, then associating with the spindle, and gradually concentrating to the spindle poles at metaphase. Upon overexpression in HeLa cells, localization of wild type WDR62 (detected by virtue of a C-terminal V5 tag) paralleled that of the endogenous protein, however, four MCPH2-associated mutant forms of WDR62 (W224S; S956CfsX38; V1402GfsX12^15^,^16^ and D955AfsX112 [this study], also carrying a V5 tag), failed to localize to the spindle poles (Supplementary Figure S10). On very rare occasions, we observed mitotic cells in which a small (not quantifiable) fraction of WDR62^W224S^ and WDR62^V1402GfsX12^ mutant proteins, otherwise diffusely distributed, localized to the poles (data not shown). These results suggested that the predominant consequence of MCPH2-associated mutations is the disruption of the dynamic shuttling and subcellular localization of WDR62 during mitosis. These observations further support the conclusion that the failure to detect WDR62 expression in the spindle poles of patient-derived *WDR62*^*exon23*^ homozygous fibroblasts indeed reflects defective shuttling of the mutant protein.

### WDR62 interacts with the Chromosome Passenger Complex

**Figure 6:**
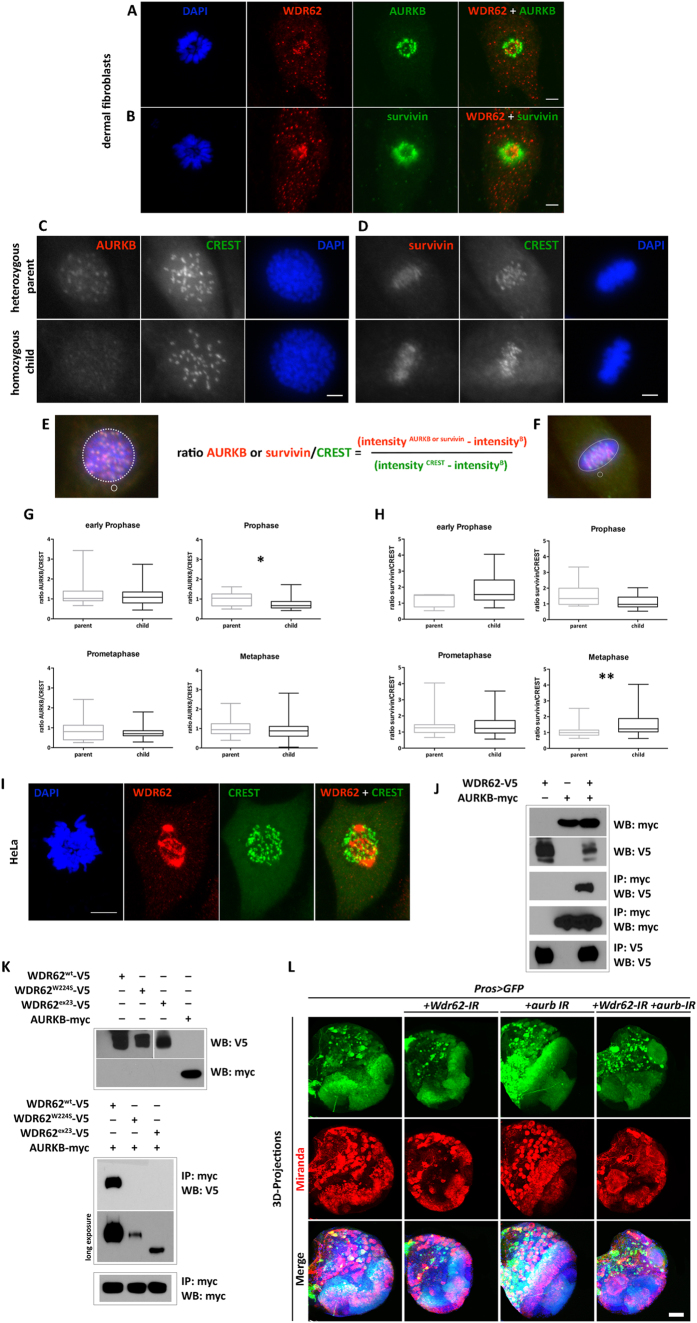
WDR62 interacts with AURKB and the Chromosome Passenger Complex. (**A,B**) Immunofluorescence staining of fibroblasts at prometaphase with antibodies specific to WDR62, AURKB, and survivin showing partial and transient co-localization of WDR62 with each protein. (**C–H**) The WDR62^exon23^ homozygous mutation affects levels of centromeric AURKB and survivin. (**C,D**) Representative images of heterozygous (top) and homozygous (bottom) fibroblasts stained with AURKB (C, prophase) or survivin (metaphase, D) (red; shown in grayscale) and FITC-conjugated CREST (green; shown in grayscale). (**E,F**) Schematic representation of method^66^ used to quantitate AURKB (**E**) and survivin (**F**) intensity. AURKB or survivin and CREST intensity was calculated by subtracting mean fluorescence intensity from a background (**B**) region (**E,F**) small circles) in the AURKB or survivin (red) and CREST (green) channel from the mean fluorescent intensity of total AURKB, survivin or CREST in the chromatin region (dotted outline on the merged images). The AURKB/CREST or survivin/CREST ratio was calculated according to the formula shown in the figure. (**G,H**) Box plot graphs of AURKB/CREST (**G**) and survivin/CREST (**H**) ratios in early prophase, prophase, prometaphase and metaphase. WDR62^exon23^ homozygous fibroblasts show significantly decreased levels of AURKB at prophase (**G**) and significantly increased levels of survivin at metaphase (H). Quantification of AURKB or survivin intensity using ImageJ was normalized to the CREST control (n = 20 to 35 cells per phase per genotype, *P < 0.05 by Mann-Whitney U test). (**I**) Immunofluorescence staining of fibroblasts shows partial and transient localization of WDR62 (red) in prometaphase kinetochores (CREST). (**J,K**) MCPH-associated mutant forms of WDR62 display reduced interaction with AURKB. Co-immunoprecipitation of WT (**J**) and mutant WDR62 (**K**) with AURKB. (**L**) Knockdown of *WDR62* and *AURKB* orthologs in *Drosophila.* Expression of *Wdr62-IR* with *prospero-Gal4* results in dramatically reduced brain size in 3^rd^ instar larvae; *aurb-IR* has no effects on brain size. Combined *Wdr62-IR* and *aurb-IR* results in formation of clusters of abnormally enlarged neuroblasts, stained with Miranda (red); nuclei are stained with DAPI (blue). Images are 3D-projection of identical Z-sections. Scale bar (**A,B**) 5 μm; (**C,D**) 50 μm; (**I**) 10 μm; (**L**) 200 μm.

To gain further insight into the function of WDR62, we interrogated transcriptomic databases of developing and adult human brain^61^ for transcripts significantly correlating with *WDR62* across developmental periods (15 total, ranging from embryonic to late adulthood) and different brain regions (transient prenatal structures and immature and mature forms of 16 brain regions, including 11 neocortical areas^61^) (Bonferoni corrected P < 0.01, n = 17,565). Functional annotation (DAVID Tools; http://david.abcc.ncifcrf.gov/62) suggested that positively correlated genes were enriched for those encoding cell cycle (Benjamini adjusted P = 7.06 × 10^−44^) and mitotic cell cycle (Benjamini adjusted P = 9.56 × 10^−40^) proteins. A group of 90 transcripts highly correlated (arbitrary correlation cutoff value 0.75) with *WDR62* (top hit; correlation value 1) (Table 1) was enriched for core components of the chromosomal passenger complex (CPC), an evolutionarily conserved regulator of several processes required for mitosis^40^. In addition to the CPC core components Aurora kinase B (AURKB), CDCA8/borealin and BIRC5/survivin (respectively regulating CPC targeting and binding to the centromere), the group also includes bona fide AURKB/CPC substrates, that are either associated with the centromere and kinetochores (SKA1, ZWINT, NCD80, DIAPH3, and CENPE) (e.g. ref. 63), or encode kinesins (e.g. KIF2C, the mitotic centromere-associated kinesin; KIF20A, a mitotic kinesin required for CPC-mediated cytokinesis; and KIF4A, a chromokinesin that suppresses microtubule dynamics and is spatially and temporally regulated by AURKB^64^). These findings prompted us to investigate WDR62 in relation to CPC components using fibroblasts and HeLa cells. During mitosis and up to metaphase, we observed that a fraction of WDR62 transiently co-localizes with AURKB (which in addition to being enriched on chromosomes, it is also targeted to the spindle where it acts on microtubule-bound substrates^65^,^66^) as well as survivin (Fig. 6A and Supplementary Figure S11). Furthermore, a fraction of WDR62 could be observed at the kinetochores (Fig. 6I). The dynamic localization of AURKB and survivin^67^ was grossly similar in heterozygous and patient-derived *WDR62*^*exon23*^fibroblasts during mitosis (data not shown). Because depletion of microtubule-associated proteins is known to affect the level of CPC accumulation at centromeres^68^, we investigated AURKB and survivin staining in fibroblasts from the affected children relative to their unaffected parents. Homozygous fibroblasts at prophase exhibited a significantly decreased level of AURKB staining at centromeres relative to heterozygous cells (p = 0.0378), as well as an increased level of survivin staining at metaphase (p = 0.0056) (Fig. 6C–H). Staining was scored at each centromere relative to the level of CREST staining for centromeric proteins^68^. These findings suggested that upon WDR62 disruption, the levels of CPC components at centromeres were perturbed, prompting us to examine whether WDR62 might physically interact with AURKB. Immunoprecipitation of HeLa cell lysates transfected with wild type WDR62 along with AURKB or survivin showed interaction with AURKB (but not with survivin) (Fig. 6J and data not shown), which was disrupted upon expression of WDR62^W224S^ and WDR62^exon23^, two MCPH2-associated mutant forms (Fig. 6K), suggesting that at least when overexpressed, the two proteins interact. Finally, we used Drosophila to examine possible genetic interactions of the fly orthologs of human *WDR62* and *AURKB*, respectively *Wdr62 (CG7337*) and *aurb*. We employed the GAL4/UAS system, using Prospero-GAL4 to drive expression of *Wdr62* and *aurb* RNAi (*Wdr62-IR* and *aurb-IR*) in neuroblasts (neural progenitor cells) and their newly born progeny (ganglion mother cells), which constitute the majority of cells in the developing larval brain^69^. *Wdr62-IR* resulted in markedly reduced brain size of 3^rd^ instar larvae as compared with controls, confirming an evolutionarily conserved role for WDR62; in contrast, *aurb-IR* had no obvious effect on brain size. Combined *Wdr62-IR* and *aurb-IR*, however, resulted in formation of clusters of abnormally enlarged neuroblasts in 40% of brains analyzed (1–6 abnormal neuroblast clusters per brain, n = 10), identified by expression of the molecular marker Miranda; the more such clusters had developed in a brain, the smaller its size (Fig. 6L and Supplementary Figure S11). These observations suggested a genetic interaction of *Wdr62 with aurb*. When considered together, these observations provide converging evidence that WDR62 can interact with AURKB.

## Discussion

In this study, we examine the effects of WDR62 disruption on neocortical development using a newly generated mouse model, identify a novel homozygous mutation in *WDR62* in two affected siblings with microcephaly and severe brain malformations, and characterize its functional impact using patient-derived fibroblasts. We establish a critical role of WDR62 in proliferation of late neocortical progenitor cells and brain size and show that its disruption causes abnormalities in asymmetric centrosome inheritance leading to neuronal migration delays, and altered neuronal differentiation. We show that mitotic progression is defective in patient-derived fibroblasts, which, similar to mouse neocortical progenitors, transiently arrest at prometaphase. We also find that expression of *WDR62* closely correlates to core components of the CPC, a major regulator of mitosis. We demonstrate that staining of CPC components at centromeres is altered in patient-derived fibroblasts and that, at least upon overexpression, mutant forms of WDR62 disrupt interactions with AURKB, the CPC enzymatic core. These findings highlight complex roles of WDR62 in mitotic cycle regulation, age-related progenitor maintenance, neuronal migration and differentiation, and may help explain the wide spectrum of structural brain abnormalities that uniquely characterize individuals with *WDR62*-associated microcephaly.

As in humans, disruption of WDR62 in mouse and Drosophila leads to microcephaly, highlighting its evolutionarily conserved function. *Wdr62*^*1-21/1-21*^mice harbor smaller brains, with significantly shorter anterior-to-posterior neocortical axis length and fewer cells overall, reflecting decreased proliferation and decreased cell cycle exit of late progenitors and increased apoptosis. WDR62 dysfunction further impacts mitotic progression, as evidenced by enrichment of cells at prometaphase in murine mitotic progenitors in the VS and in dividing fibroblasts from individuals with MCPH2. In the murine neocortex, WDR62 loss disrupts asymmetric centrosome inheritance, a mechanism that has been previously linked to progenitor maintenance^44^. Disruption of centrosome inheritance also causes an imbalance in the behavior of newly postmitotic neurons, which, instead of migrating away, preferentially remain within the proliferating zones (Fig. 3). The underlying mechanism remains unknown, but the Drosophila *Wdr62* ortholog also plays a role in maintaining centrosome asymmetry in neuroblasts and is required for microtubule stabilization and maintenance of microtubule organizing center activity on the apical interphase centrosome^70^. WDR62 disruption in patient-derived fibroblasts has no obvious impact on spindle or centrosome formation and proper localization of many pericentrosomal and centrosomal proteins; the localization of (endogenous or overexpressed) disease-associated mutant forms of WDR62 to the spindles poles, however, is impaired.

Previous studies also suggested that knockdown^35^ or genetic inactivation of WDR62^34^ leads to depletion of neural progenitors. Our findings in the *Wdr62*^*1-21/1-21*^mouse partially confirm those from the *Wdr62*^*1-14/1-14*^ mouse in which *Wdr62* is disrupted after exon 14^34^; differences in insertion sites possibly account for the phenotypic discrepancies. Increased cell death and preferential loss of late neocortical progenitors occur in both models; however, we also uncovered a decrease in mitotic index and cell cycle exit as well as defects in neuronal differentiation manifested after birth, with several subtypes being differentially affected (Fig. 4). WDR62 disruption leads to delayed mitotic progression and prometaphase/metaphase arrest in *Wdr62*^*1-14/1-14*^MEFs^34^ and we found that *Wdr62*^*1-21/1-21*^ neocortical progenitors and patient-derived fibroblasts also transiently arrest in prometaphase. While *Wdr62*^*1-14/1-14*^MEFs commonly display multipolar spindles, extra centrosomes and lagging chromosomes^34^, *WDR62*^*exon23*^homozygous fibroblasts harbor extra centrioles only very rarely (Supplementary Figure S8) and lack lagging chromosomes or multipolar spindles, suggesting that the domain encompassing WD repeats 11–13, encoded by exons 15 to 23, which is maintained in human, but not mouse fibroblasts, may be critical for spindle and centrosome assembly. It is known that prolonged prometaphase blocks daughter cell proliferation in human fibroblasts, despite normal completion of mitosis^71^ and mitotic delay correlates with preferential production of neurons at the expense of neural progenitors, as well as of apoptotic progeny in mouse neocortex^50^. Furthermore, disruption of normal cell cycle progression is known to cause apoptosis^72^ and abnormal cortical development^46^,^47^,^48^,^50^,^73^. Therefore, the documented and complex effects that WDR62 disruption has on the mitotic cycle of neural progenitors could explain the microcephaly with structural abnormalities in patients with MCPH2.

WDR62 disruption differentially impacts neural progenitors, with more pronounced effects in late neurogenesis. We did not examine whether all progenitors are equally affected at a given time, or whether the fraction of those affected eventually reaches a threshold. However, it is possible that even among progenitors at a given developmental stage, the effect of WDR62 is different, depending on their inherent proliferation properties. It is noteworthy that haploinsufficiency of the exon junction complex component Magoh only affects a subset of progenitors^50^, suggesting progenitor heterogeneity^74^,^75^. Pilaz *et al*.^50^ speculate that an internal clock capable of measuring mitosis duration may exist in neural progenitors; such clock may be differentially regulated during neurogenesis, especially in light of the observation that many genes whose expression highly correlates with *WDR62* (Supplementary Table S1) display circadian expression pattern and have been implicated in transcriptional coordination of mitosis, albeit in a non-neural system^76^.

WD40 repeat-containing proteins coordinate multiprotein complex assemblies^18^,^19^. WDR62 interacts with JNK1/2 and Aurora A (AURKA) kinases, which, respectively, negatively regulate its association with microtubules, and control its localization to the spindle poles^36^,^37^,^38^,^39^. *In vivo*, WDR62 interacts genetically and physically with AURKA, and also associates with the mitotic spindle regulator TPX2^34^,^77^. Furthermore, WDR62 and several other MCPH-associated proteins assemble at the centrosome where they promote centriole duplication^43^. We now demonstrate that WDR62 can interact with AURKB, the CPC core enzyme. It is known that depletion of any CPC component disrupts mitotic progression^40^,^78^,^79^,^80^, and WDR62 disruption causes a modest decrease in kinetochore levels of AURKB, and a significant increase in kinetochore levels of survivin in patient-derived fibroblasts. Although individual subunits of the CPC protein complex usually co-vary in this analysis^68^, it is possible that depletion of functional WDR62 has different effects on CPC subunits. The abnormalities in the AURKB and survivin relative ratios in *WDR62*^*exon23*^ homozygous fibroblasts suggest perturbed kinetochore function, especially as *Wdr62*^*1-14/1-14*^MEFs were reported to exhibit increased tension across sister kinetochores^34^. In this regard, it is noteworthy that the kinetochore-associated genes *CASC5*^81^ and *CENPE*^82^ have been associated with MCPH and that disruption in kinetochore localization is associated with microlissencephaly^83^ and that lissencephaly is a common comorbidity in MCPH2. Lastly, AURKB has been linked to embryonic stem cell pluripotency through phosphorylation-mediated inhibition of the master transcription factor OCT4^84^. Whether AURKB kinetochore levels are also perturbed in neural progenitors upon WDR62 disruption is not known, and if this could have an effect on mitotic phosphorylation of transcription factors ultimately affecting cell fate remains to be investigated in future studies.

Our findings demonstrate complex effects of MCPH2-associated mutant forms of WDR62 on mitotic cycle progression, control of asymmetric centrosome inheritance and its association with the CPC, highlighting critical roles for WDR62 in brain morphogenesis and disease pathogenesis.

## Methods

### WDR62^*1-21/1-21*^mice

*Wdr62*^*1-21/1-21*^ mice were generated from trapped ES cells (Wdr62^Gt(CH0428)Wtsi^) obtained from the Welcome Trust Sanger Institute (www.informatics.jax.org) and maintained in accordance with the National Institutes of Health guidelines and approval of the Yale University Institutional Animal Care and Use Committee.

### Brain fixation

Embryonic brains (except E18.5) were extracted and immersed in 4% paraformaldehyde (PFA) in 0.1 M phosphate buffer (PBS) for 24 hours and up to several days, and processed, respectively, for immunohistochemistry and *in situ* hybridization or Nissl staining. Brains of E18.5 embryos, neonatal and postnatal mice were extracted after transcardiac perfusion with cold PBS followed by 4% PFA.

### Brain measurements and quantification of cortical cell number

Brains were harvested at the indicated stages and fixed as described above. Measurements (in mm) were obtained along the anterior-most to posterior part of the forebrain (A-P axis length) and at the widest part of the forebrain (M-L axis length), as indicated in Fig. 1, using a Jameson Caliper.

Brains of 9-week-old mice were harvested and post-fixed by immersion in 4% PFA for 6–8 weeks. The cerebral cortex and the hippocampus were dissected and total cell numbers were estimated using the isotropic fractionator as previously described^41^. Briefly, samples were mechanically dissociated in a solution containing 40 mM sodium citrate and 1% Triton-X, turned into an isotropic suspension of isolated nuclei, and kept homogenous by agitation. The total number of nuclei in suspension, and therefore, the total number of cells in the original tissue, was estimated by determining the density of nuclei in small aliquots stained with DAPI, under the microscope with a 40X objective, using a hemocytometer for quantification.

### Histological analyses and X-gal histochemistry

Brains at the indicated stages were fixed, extracted, and sectioned with a sledge cryomicrotome (Leica Microsystems, Wetzlar, Germany). Nissl staining was performed on serial sections by standard procedures. Embryonic brain sections were stained for β-galactosidase activity in a solution containing 0.5 mM potassium ferricyanide, 0.5 mM potassium ferrocyanide, 20 mM magnesium chloride, 0.1% Triton X-100, and 0.37 mg/ml X-gal.

### BrdU labeling

Pregnant females were injected intraperitoneally with a solution of 5-bromo-2′-deoxyuridine (BrdU; 10 mg/ml in saline) at 20 μg/g of body weight and/or with 5-chloro-2′-deoxyuridine (CldU; 10 mg/ml in saline) at 42.5 μg/g of body weight. Brains were harvested and processed for immunohistochemistry or immunofluorescence with antibodies specific to BrdU and/or CldU.

### TUNEL assay

Brain sections (36 μm thickness) were obtained with a cryomicrotome (Leica), air-dried, post-fixed in 4% PFA for 15 min, washed with PBS, incubated with Proteinase K (1 μg/ml) in Proteinase K buffer (100 mM Tris-HCl, pH 8.0, 50 mM EDTA pH 8.0) at 37 °C for 30 min, washed with PBS and processed using the Millipore S7100 apoptosis detection kit (Fisher Scientific) following the manufacturer’s instructions.

### *In situ* hybridization

Embryonic and postnatal brains were fixed, respectively, by immersion in or transcardiac perfusion with 4% PFA, cryoprotected in 30% sucrose (in 4% PFA) and sectioned with a cryomicrotome (Leica). Human tissue was obtained from several sources, including the Human Fetal Tissue Repository at the Albert Einstein College of Medicine (New York, NY), fixed by immersion in 4% PFA and sectioned. Sections were processed for *in situ* hybridization as described previously^85^. RNA probes complementary to mouse transcripts were obtained from colleagues [*Pax6*^86^, *Tbr2*^87^, *Cux2*^88^,^89^), *Er81* (a.k.a. *Etv1, Mouse Genome Informatics*^90^, *Tle4*^91^, *claudin 11* (gift from S. Tsukita, Kyoto University, Japan), *Notch1, reelin, Scip* (gifts from E. Grove, University of Chicago), *Rorb* (gift from C. Ragsdale, University of Chicago)], or generated using oligonucleotide pairs as indicated below (for *Wdr62,* see ref. 15). cRNAs were labeled with digoxigenin-11-UTP. Sections were analyzed using a Stemi stereomicroscope or an AxioImager (Zeiss, Oberkochen, Germany) fitted with an AxioCam MRc5 digital camera. Images were captured using AxioVision software (Zeiss) and assembled in Adobe Photoshop.

Oligonucleotides used to generate the *in situ* probes as shown in Table 2.

### Immunofluorescence - brain sections

For immunofluorescence staining of sections at prenatal stages, brains were harvested and fixed as described above. The brains were cryoprotected by sequential immersion in 15% and 30% sucrose in PBS, flash frozen into embedding medium (Tissue-Tek, Sakura Finetek USA, Torrance, CA) and sectioned using a cryostat (Leica). Sections (12 μm) were air-dried, post-fixed in 4% PFA for 10 min and washed with PBS 3 times for 5 min each, blocked in a solution containing 5% normal donkey serum (Jackson ImmunoResearch Laboratories, West Grove, PA), 1% bovine serum albumin, 0.1% glycine, 0.1% L-lysine supplemented with 0.3% Triton X-100 for 1 hour and then incubated in the above solution with the appropriate primary antibody overnight at 4 °C. Sections were washed 3 times in PBS for 10 min each and incubated with secondary antibodies [Alexa-Fluor conjugated (Molecular Probes; 1:300 dilution)] in blocking solution without detergent for 2 hours at room temperature. For immunofluorescence staining at postnatal stages, brains were harvested following transcardiac perfusion as described above, post-fixed in 4% PFA overnight and cryoprotected in 30% sucrose in PBS for several hours. Sections (50 μm) were obtained on a cryomicrotome (Leica) and processed free-floating. After washing 2 times in PBS, 5 min each, sections were incubated in blocking solution (same as above) with 0.9% Triton X-100 for 1–2 hours at room temperature, followed by incubation with the appropriate primary antibodies in the same blocking solution for 72 hours at 4 °C. Sections were washed 3 times with PBS, 15 min each and incubated with the appropriate secondary antibodies in blocking solution without detergent for 2 hours at room temperature.

### Immunofluorescence – cells

Human fibroblasts, iPSCs, murine embryonic cortical neurons and HeLa cells were fixed with cold 4% PFA for 15 min at room temperature, washed 3 times with PBS, incubated in blocking solution (same as above) for 1 hour and then in primary antibody in blocking solution for 1 hour at room temperature. After 3 washes with PBS, secondary antibodies were applied in blocking solution without detergent for 1 hour at room temperature. Samples were mounted with Vectashield containing DAPI (Vector Laboratories), and imaged with a Zeiss LSM or a Leica TCS SP2 laser scanning confocal microscopy system.

### Antibodies

WDR62 (Bethyl Laboratories, #301–560, 1:500); BrdU (Becton Dickinson, #BD-347580, 1:100; abcam, #ab1893, 1:100), CldU (Accurate Scientific, OB T00 30 G, 1:100), Ki67 (Thermo Scientific, SP6, RM-9106-S0, 1:200), SATB2 (Genway, #GWB-9F2D2F, 1:200), CTIP2 (abcam, #ab18465, 1:250), TBR1 (Santa Cruz, #sc-48816, 1:250), WDR62 (Bethyl Lab, #A301–560A, 1:500), gamma-tubulin (Sigma, #T6557, 1:500), ASPM (Novus Biologicals, #NB100–2278, 1:500), AIM-1 (Aurora kinase B; #BD-611083, 1:300), CEP63 (ED Millipore, #06–1292, 1:500), CEP135 (abcam, #ab75005, 1:1000), PH3 (Cell Signaling, #9701 S, 1:300), CDK5RAP2 (Bethyl, #IHC-00063, 1:100), BIRC5/survivin (Santa Cruz, #sc-17779, 1:100), CREST (Human anti-Centromere Proteins; Antibodies Incorporated, #15–235-F, 1:100); myc (for immunoprecipitation: rabbit polyclonal abcam #9106; for Western blot: monoclonal 9E10, 1:1,000); V5 (monoclonal TCM5, eBioscience #14–6796; for Western blot: 1:1,000; for immunofluorescence 1:500); V5 (polyclonal Millipore #AB3792 for immunofluorescence 1:600).

### *In utero* electroporation and photoconversion

All surgical procedures were performed using sterile conditions and in accordance with IACUC approved protocols. *In utero* electroporation was performed using standard procedures^92^ and the photoconversion was performed as previously described^44^. The Kaede-centrin1 plasmid was a gift from S.-H. Shi (Memorial Sloan Kettering Cancer Center, New York).

### Primary cultures of embryonic cortical neurons

Cortical leaves from E15.5 embryos were dissected in cold Hanks’ Balanced Salt Solution (HBSS) supplemented with 0.5% D-glucose and 25 mM HEPES (HBSS+). Minced cortical tissue samples were dissociated using Papain-Protease-DNAse I (PDD) solution (190 μl Papain, 50 mg Dispase II and 5 mg DNAse I per 50 ml of HBSS+) and incubated at 37 °C for 30–40 min. Samples were subjected to centrifugation at 500 g for 4 min and pellets were resuspended in 1 ml of Neurobasal medium supplemented with 1 mM Sodium Pyruvate, 2 mM Glutamax, Pen/Strep and B-27 Supplement and filtered through a 40-micron mesh filter. Cells were plated on glass coverslips coated with laminin and poly-ornithine and cultured in Neurobasal medium as above.

### Skin biopsy and fibroblast culture

A 4-mm skin punch biopsy was taken under local anesthesia from the umbilical area of NG1406-1, NG1406-2, and their parents (NG1406-3, 1406-4) using standard procedures^93^,^94^. Samples were stored in Dulbecco’s Modified Eagle Medium (DMEM; Gibco, #11965-084) supplemented with 10% heat-inactivated fetal bovine serum (FBS; Gibco, #10438-026), 1% (1x) L-glutamine (Gibco, #25030-081), 2% (1x) Penicillin-Streptomycin (Gibco, #15140-122) and processed immediately on arrival. Following wash with PBS, the samples were minced and small fragments were laid onto the surface of 100 mm^2^ Petri dishes, in square areas marked by perpendicular lines made with scalpel blades. The fragments were allowed to air dry so as the dermis side of specimens adhered to the culture dish. The fragments were then cultured in DMEM with 10% FBS, 1% (1x) L-glutamine, 1% (1x) Penicillin-Streptomycin at 37 °C. The fibroblasts typically grew within 7–9 days.

### Cell culture and synchronization

Human fibroblasts and HeLa cells were maintained in DMEM supplemented with 10% fetal bovine serum. To synchronize the fibroblast cultures, cells were rinsed with PBS and grown in serum-free medium for 18 hours, then seeded in gelatin-coated chamber slides and released into the cell cycle for 36 hours by addition of serum prior to fixation with 4% PFA and analysis. For arrest in prometaphase, asynchronous fibroblast cultures were treated with 0.2 μg/ml nocodazole (Sigma) for 14 hours, then fixed for analysis. Primary fibroblasts synchronized in prometaphase were washed and released into growth medium for 1 hour, fixed, and analyzed for progression to metaphase.

### Nucleofection of fibroblasts

Fibroblasts were harvested by centrifugation and processed for transfection using a Nucleofector 2b device (Lonza, Allendale, NJ) and a kit for Human Dermal Fibroblasts (NHDF; Lonza) according to the manufacturer’s instructions. Cells (5 × 10^5^) were nucleofected with 3 micrograms of Kaede-CETN1 plasmid^44^, plated on gelatin-coated chamber slides and exposed to violet light 24 hours later in order to photoconvert the fluorescent Kaede-CETN1 fusion protein. 48 hours after photoconversion, the cells were fixed and imaged with confocal microscopy (Leica DM6000 CFS). Images were processed using ImageJ and centrosomes were counted using Adobe Photoshop.

### Immunoprecipitation

HeLa cells were cultured in DMEM with 10% fetal calf serum and transiently transfected with V5-tagged WT or mutant forms of WDR62 along with AURKB-3xMyc using Lipofectamine (Life Technologies, NY). 36 hours after transfection cells were treated with 0.2 μg/ml nocodazole (Sigma) for 12 hours, then were lysed using a Triton-X100 based buffer containing protease and phosphatase inhibitor cocktails (Calbiochem, San Diego, CA). Clarified supernatants were used for immunoprecipitation using Dynabeads (Life Technologies) according to the supplier’s instructions. Immunoprecipitates were analyzed by Western analysis using standard protocols. In brief, lysates were separated on gradient SDS-PAGE gels (4–16%, BioRad) and transferred to nitrocellulose or PVDF membranes. After blocking with 5% milk, the membranes were incubated first with rabbit anti-tag antibodies and then with a secondary HRP-conjugated anti-rabbit IgG (Jackson Immunoresearch Labs). Signals were detected with chemiluminescence reagent (BioRad).

### Quantitative Analysis

All data obtained were analyzed using two-tailed Student’s *t*-test or Mann-Whitney U test with a significance level of at least P < 0.05 (^★^p < 0.05, ^★★^p < 0.01, ^★★★^p < 0.005). Values designate mean; error bars represent s.e.m.

### Layer distribution analysis

To quantify the distribution of neurons, anatomically matched sections were selected. The neocortex of P3 (SATB2 immunofluorescence) or E18.5 brains (Kaede-CETN1 expression) was subdivided radially from the pia surface to the upper edge of the white matter, into 10 or 3 equally sized bins, respectively. SATB2^+^ cells or labeled centrosomes were counted in each bin as indicated in the respective graphs.

### Imaging

Visualization of stained brain sections or cells was performed using a Stemi stereomicroscope or an AxioImager (Zeiss) fitted with an AxioCam MRc5 digital camera, or using a TCS SP5 confocal microscope (Leica). Images were captured using the AxioVision (Zeiss) or the integrated LAC (Leica) software. Confocal images were assembled using LAS AF lite (Leica Application Suite, Leica) or Image J (developed by Wayne Rasband, National Institutes of Health; http://imagej.nih.gov/ij). All images were processed with Image J and/or Adobe Photoshop.

### Cloning and mutagenesis

Cloning of all genes in expression vectors was performed using the Gateway system (Life Sciences). Gateway BP and LR reactions were performed using BP and LR clonase, respectively, according to the vendor’s instructions. PCR reactions to amplify genes and/or gene fragments were performed using the high-fidelity Accuprime Pfx DNA polymerase (Life Sciences) while all mutagenesis reactions were performed using the high-fidelity Phusion DNA polymerase (New England Biolabs). Gateway B1/B2 PCR reactions were performed using primers below, in order to amplify the cDNA of interest. To design primers, we used the QuickChange Primer Design website (Agilent Technologies, Inc). B1/B2 PCR products containing the cDNA of the desired gene were purified using the PCR purification kit (Qiagen) and were recombined using BP clonase into the shuttle plasmid pDONR-zeo (Life Sciences). This yielded so-called cDNA ENTRY clones. All genes were fully sequenced at this stage. When needed, site-directed PCR mutagenesis was performed on cDNAs ENTRY clones, then fully sequenced. Once the sequence of any ENTRY clone was confirmed, the respective plasmid DNA was recombined using LR clonase into a common, CMV promoter-driven expression vector, pCDNA-DEST40 (Life Sciences), which carries a V5-HIS tandem tag at the 3′ end of the Gateway cassette. All B2 primers depicted below have no STOP codon, allowing tagging of all genes with the V5-HIS tandem tags present in pCDNA-DEST40.

pCAN3Myc-WDR62 (encoding the CS5 transcript of WDR62) was a gift from A. Aronheim (The Rappaport Faculty of Medicine, Technion, Israel)^38^. This cDNA was modified to include an in-frame 194 bp fragment that is present in the brain-enriched transcript (consensus WDR62 sequence, GenBank). To clone this fragment, the missing sequence was amplified from a fetal brain cDNA library using the primers below and the PCR product was inserted in the *WDR62* cDNA sequence via standard PCR mutagenesis using Phusion polymerase.

Four mutations described in patients with MCPH2 were introduced in the wild type *WDR62* cDNA. The same mutagenesis protocol and the respective primers (see below) were used to introduce the W224S missense mutation. To generate the shorter, truncated versions of WDR62 mutations were introduced in exon 23 (Asp955AlafsX112), intron 23 (S956CfsX38), and exon 31 (V1402GfsX12), using Gateway with reverse (B2) primers targeting the respective regions. Since the GACA deletion in exon 23 causes a frameshift that adds 112 amino acids until the next “stop” codon, we used PCR insertional mutagenesis to introduce the 112-amino acid tail to the previously made exon 23 deletion construct. The above PCR product obtained with the W62xMF/MR primer pair was purified and used for mutagenesis to insert the 112-amino acid tail “in-frame” at the end of the construct previously generated with the B2nsEX23 W primer. In all constructs the STOP codons at the end of the ORF were deleted to allow 3′ tagging of the respective cloned genes with the V5-His tandem peptide tags present within the DEST40 plasmid.

A full-length cDNA encoding Aurora Kinase B was purchased from Addgene (plasmid 23682: pDONR223-AURKB). The gene was recombined via LR reactions into the pcDNADEST40 Gateway expression vector. In addition, the V5-His tag was removed and replaced with a 3x Myc tag.

All constructs generated were confirmed by Sanger sequencing.

Gateway oligonucleotides used to subclone the *WDR62* full length gene:

B1-W62: GGGGACAAGTTTGTACAAAAAAGCAGGCTTCGCCACCATGGCGGCCGTAGGGTCCGGAGGCTATG

B2nsW62: GGGGACCACTTTGTACAAGAAAGCTGGGTCCCAGTGCCCCCGTGCCTTCCTCCGCAC

Oligonucleotides used to insert the 194 bp fragment into the original clone:

F: GTGCTGGAGAAGTGGATCAACCTGAAG

R: GTCGAAGGTCAGTGCCACTGTATCTGG

Gateway oligonucleotides used to subclone WDR62 truncated proteins:

B2nsEX31W: GGGGACCACTTTGTACAAGAAAGCTGGGTCCCAGGGCTCACTCCAGCGGGCAGGGCTGCCCTGTAACA

B2nsEX23W: GGGGACCACTTTGTACAAGAAAGCTGGGTCGCTGTCTGTCCCTGTGACTGTCACTTC

Oligonucleotides used to perform the W224S mutagenesis

W224SF: CACTGTTGGGAACCGCCATGTGAGGTTCTCGTTCTTGGAAGTCTCCACTGAGACAAAG

W224SR: CTTTGTCTCAGTGGAGACTTCCAAGAACGAGAACCTCACATGGCGGTTCCCAACAGTG

Oligonucleotides used to add the “tail” of 112 amino acids in the exon 23 construct

W62 × 22MF: TACTCTCTGGAGGCAGAAGTGACAGTCACAGAGACAGCCAGTATTGCAGGAAGGAGGTG

W62 × 22MRns: AACTTTGTACAAGAAAGCTGGGTCGTGTCTCAAAGTGGTGGCGGAGGAACTTCT

### Human subjects

The study protocol was approved by the Yale Human Investigation Committee (HIC) (IRB #9406007680). Institutional review board approvals for genetic and MRI studies, along with written informed consent from all study subjects, were obtained by the referring physicians at the participating institutions. Human fetal brain specimens at 19 weeks of gestation were obtained from the Human Fetal Tissue Repository at the Albert Einstein College of Medicine (CCI number 1993–042). Postmortem human brain specimens were obtained from tissue collections at the Department of Neuroscience at Yale School of Medicine.

### Clinical histories of patients (NG1406 family)

The index case (NG1406-1) is a 13-year-old full term male who is the product of a second-cousin marriage. He presented for medical attention at 20 months of age owning to global developmental delay and was found on clinical examination to have microcephaly and dysmorphic facies. Neuroimaging studies identified an array of developmental abnormalities including diffuse pachygyria, thickened cortex and hypoplasia of the corpus callosum and three-dimensional computed tomography showed metopic synostosis. At 8 years of age, he appeared microcephalic (head circumference: 44.5 cm, <2^nd^ centile; height: 116 cm, 3^rd^–10^th^ centile; weight: 20 kg; 3^rd^–10^th^ centile). He never developed the ability to speak. Large auricles were noted. The index case’s 6-year-old brother (NG1406-2) displayed similar symptoms. He was brought to medical attention as soon as he was born with microcephaly (head circumference: 31 cm, <2^nd^ centile). He developed head control at 3 months and ability to sit without support at 7 months of age. Global developmental delay was detected with the DENVER developmental screening test, performed at 18 months. At age 2 years, head circumference was 41 cm (<2^nd^ centile), height was 83 cm (10^th^–25^th^ centile) and weight was 10 kg (<3^rd^ centile). General examination was remarkable for global developmental delay, no ability to walk, hypoplastic forehead and large auricles. At age 3 1/2, head circumference was 46.5 cm (<2^nd^ centile), height was 129 cm (10^th^–25^th^ centile) and weight was 22 kg (<3^rd^ centile). Like his brother, he exhibited microcephaly and no ability to speak fluently. Their routine blood studies, including neurometabolic assays, complete metabolic panel, complete blood count, and thyroid function tests were all within normal limits. Whole exome sequencing of the younger affected brother identified a novel homozygous frameshift mutation, D955AfsX112, caused by a 4 bp deletion in exon 23 of *WDR62*, resulting in a premature stop codon at position 1067; the mutation was confirmed to be homozygous in both affected subjects, and heterozygous in both parents, using Sanger sequencing. It was not observed in more than 1,800 Turkish control chromosomes, or in the ESP (exome sequencing project) and 1,000 genome databases.

### Whole-exome capture and sequencing

Exome capture for the index case was performed using the NimbleGen 2.1 M human exome array (Roche Nimblegen, Inc., Madison, WI, USA) according to the manufacturer’s protocol along with modifications previously described^15^,^95^. Exome library sequencing was performed using the HiSeq2000 with barcoding technology, paired end analysis and six samples per lane. Image analysis and subsequent base calling were performed using the Illumina pipeline (version 1.8, Illumina, Inc.).

### Exome data analysis

Analysis of the sequencing data was performed according to the previously described bioinformatics pipeline devised by our research team^96^.

### Sanger sequencing

The mutation was evaluated by Sanger sequencing using standard protocols. Amplicons were cycle sequenced on ABI 9800 Fast Thermo cyclers (Applied Biosystems, Foster City, CA, USA), and post cycle sequencing clean-up was carried out with the CleanSEQ System (Beckman Coulter Genomics, Danvers, MA, USA). The amplicons were analyzed on a 3730 Å~L DNA Analyzer (Applied Biosystems Inc.).

## Additional Information

**How to cite this article:** Sgourdou, P. *et al*. Disruptions in asymmetric centrosome inheritance and WDR62-Aurora kinase B interactions in primary microcephaly. *Sci. Rep.* **7**, 43708; doi: 10.1038/srep43708 (2017).

**Publisher's note:** Springer Nature remains neutral with regard to jurisdictional claims in published maps and institutional affiliations.

## Supplementary Material

Supplementary Information

## Figures and Tables

**Table 1:** Co-expression analyses of *WDR62* in developing and adult human brain.

ID	Gene Symbol	RefSeq	Correlation	Function
3831062	WDR62	NM_001083961	1	
3413875	TROAP	NM_005480	0.853213572	Spindle assembly, MT associated
3235789	MCM10	NM_182751	0.844120137	DNA replication
3415857	ESPL1	NM_012291	0.842984542	Sister chromatid segregation
3590086	RAD51	NM_002875	0.839212882	Mitotic recombination
3203935	KIF24	NM_194313	0.835263352	Centriolar kinesin
2438282	IQGAP3	NM_178229	0.835156551	Cell proliferation
3204692	CCDC107	NM_174923	0.822303373	
3365776	E2F8	NM_024680	0.821666086	Cell proliferation; cell cycle progression
3457824	TIMELESS	NM_003920	0.817993637	DNA replication
3936913	CDC45L	NM_003504	0.817924629	DNA replication
3689880	SHCBP1	NM_024745	0.813831525	
2334098	KIF2C	NM_006845	0.810306051	Centromere-associated kinesin
3463112	E2F7	NM_203394	0.809088244	Cell cycle progression
2388219	EXO1	NM_130398	0.804499522	DNA repair
2691575	POLQ	NM_199420	0.803547962	DNA polymerase
3949055	GTSE1	NM_016426	0.803194446	MT-associated
2610241	FANCD2	NM_033084	0.803078959	DNA damage repair
3090697	CDCA2	NM_152562	0.802514482	Mitotic exit
3607698	C15orf42	NM_152259	0.800566075	Cell cycle machinery
3168508	MELK	NM_014791	0.800534753	Cell cycle regulation
3788049	SKA1	NM_001039535	0.799294112	Kinetochore MT-associated
2571457	CKAP2L	NM_152515	0.79841561	Mitotic spindle
2330773	CDCA8	NM_018101	0.797718577	Borealin; CPC component
4052881	FAM72D	AB096683	0.797179155	
2604254	HJURP	NM_018410	0.796087409	Centromere; chromatin assembly factor
2412799	ORC1L	NM_004153	0.795398232	DNA replication
2450345	KIF14	NM_014875	0.795334068	Mitotic kinesin
3736290	BIRC5	NM_001168	0.794444828	Survivin; CPC component
2406420	CLSPN	NM_022111	0.793740153	ATR checkpoint mediator
2714955	TACC3	NM_006342	0.792847226	Mitotic spindle
3493391	C13orf34	NM_024808	0.791809623	Mitotic entry
3440598	FOXM1	NM_202002	0.791732636	Cell cycle progression
3484641	BRCA2	NM_000059	0.789600978	Hom. recombination; DNA damage repair
2494484	NCAPH	NM_015341	0.789048384	Chromatin condensation
3290210	ZWINT	NM_032997	0.787588546	Kinetochore protein
3258444	CEP55	NM_018131	0.786424301	Centrosomal protein
2640855	MCM2	NM_004526	0.785821418	DNA replication
3776139	NDC80	NM_006101	0.785774461	Kinetochore protein
3515965	DIAPH3	NM_001042517	0.785738543	MT dynamics
3884892	FAM83D	NM_030919	0.785094043	Mitotic chromosome alignment
3744263	AURKB	NM_004217	0.783021852	CPC component
3599811	KIF23	NM_138555	0.781181975	Mitotic spindle
3886223	MYBL2	NM_002466	0.780423465	Cell cycle progression regulator
3726375	EME1	NM_152463	0.778537537	DNA repair
3758317	BRCA1	NM_007300	0.77847807	DNA repair
3401804	RAD51AP1	NM_001130862	0.777301727	Genomic integrity
2899772	HIST1H2AH	NM_080596	0.777262236	Histone
3750785	SPAG5	NM_006461	0.776919588	Mitotic spindle; MT-associated
2454444	NEK2	NM_002497	0.77517357	Centrosomal protein
3850660	SPC24	NM_182513	0.774731925	NDC80 complex component
3962165	CENPM	NM_024053	0.773822569	Kinetochore protein
3608298	BLM	NM_000057	0.773651449	RecQ helicase
2364438	NUF2	NM_145697	0.773491393	NCD80 complex component
3565663	DLGAP5	NM_001146015	0.770357896	Potential cell cycle regulator
2830638	KIF20A	NM_005733	0.768855545	MT-associated
3980560	KIF4A	NM_012310	0.767678415	Chromokinesin
3158767	RECQL4	NM_004260	0.767437811	Telomere maintenance
3556990	JUB	NM_032876	0.767143488	Centrosome and kinetochores
2665572	SGOL1	NM_001012410	0.766632082	Centromeric protein
3720896	CDC6	NM_001254	0.766242562	DNA replication and spindle formation
2838656	HMMR	NM_001142556	0.766233469	BRCA1 and BRCA2 complex
4012142	ERCC6L	NM_017669	0.764570362	DNA helicase; SAC
2780172	CENPE	NM_001813	0.763971047	Kinetochore protein
2673085	CDC25A	NM_001789	0.763279202	Cell cycle and apoptosis regulator
3428845	C12orf48	AK302724	0.762213341	DNA repair
3367338	KIF18A	NM_031217	0.762065719	Chromosome positioning; spindle length
3331903	FAM111B	NM_198947	0.760818719	
3722770	C17orf53	AK291924	0.760631969	
2469252	RRM2	NM_001034	0.759932681	Ribonucleotide reductase subunit
3619945	OIP5	NM_007280	0.759425743	Centromeric protein
3821701	ZNF788	NR_027049	0.758954541	
3607537	FANCI	NM_001113378	0.758907703	Genomic stability
2748163	MND1	NM_032117	0.754467347	Meiotic recombination
2675936	WDR51A	NM_015426	0.754397251	Centriolar protein; centrosome
3883941	TGIF2	NM_021809	0.754154872	Transcriptional co-repressor
2914777	TTK	NM_003318	0.753919292	Kinetochore protein
3607510	FANCI	NM_001113378	0.7538568	Genomic stability
2902178	TCF19	NM_007109	0.753793898	G1/S transition
3706753	GSG2	NM_031965	0.753512776	Haspin; CPC regulator
2877378	CDC25C	NM_001790	0.753298991	Cell cycle control; mitotic entry
2401448	E2F2	NM_004091	0.75215418	Cell cycle regulator
3629103	KIAA0101	NM_014736	0.752038592	Centrosomal protein
2585933	SPC25	NM_020675	0.751330344	NDC80 complex component
3129149	PBK	NM_018492	0.750144834	Mitosis

**Table 2:** Oligonucleotides used to generate the in situ probes.

Gene	Forward primer	Reverse primer
*Aspm*	ATGGCGGCCACACTTATTGA	TCCGGAAAGTGGCCTGAATC
*Glast*	AGAGATTGCAGCAAGGGGTC	AGAGATTGCAGCAAGGGGTC
*Satb2*	GTCTCCTCATAGCCAAATCCAC	GTGGTACCTCTGGTTCTGGAAG
*Sox2*	CCAAGATGCACAACTCGGAG	TACCTCTTCCTCCCACTCCA
