# Supplementary material for: Disruptions in asymmetric centrosome inheritance and WDR62-Aurora kinase B interactions in primary microcephaly
Source: Sci Rep. 2017 Mar 8;7:43708. doi: 10.1038/srep43708 (PMC5341122; doi:10.1038/srep43708)
Supplement: Supplementary Information [file srep43708-s1.pdf]

Supplementary Information – Sgourdou et al.

Disruptions in asymmetric centrosome inheritance and WDR62-Aurora kinase B interactions in primary microcephaly

Paraskevi Sgourdou[1], Ketu Mishra-Gorur[1], Ichiko Saotome[1], Octavian Henagariu[1], Beyhan Tuysuz[2], Cynthia Campos[1], Keiko Ishigame[1], Krinio Giannikou[1], Jennifer L. Quon[1], Nenad Sestan[3], Ahmet O. Caglayan[1,4], Murat Gunel[1,5], Angeliki Louvi[1,*]

FIG. S1

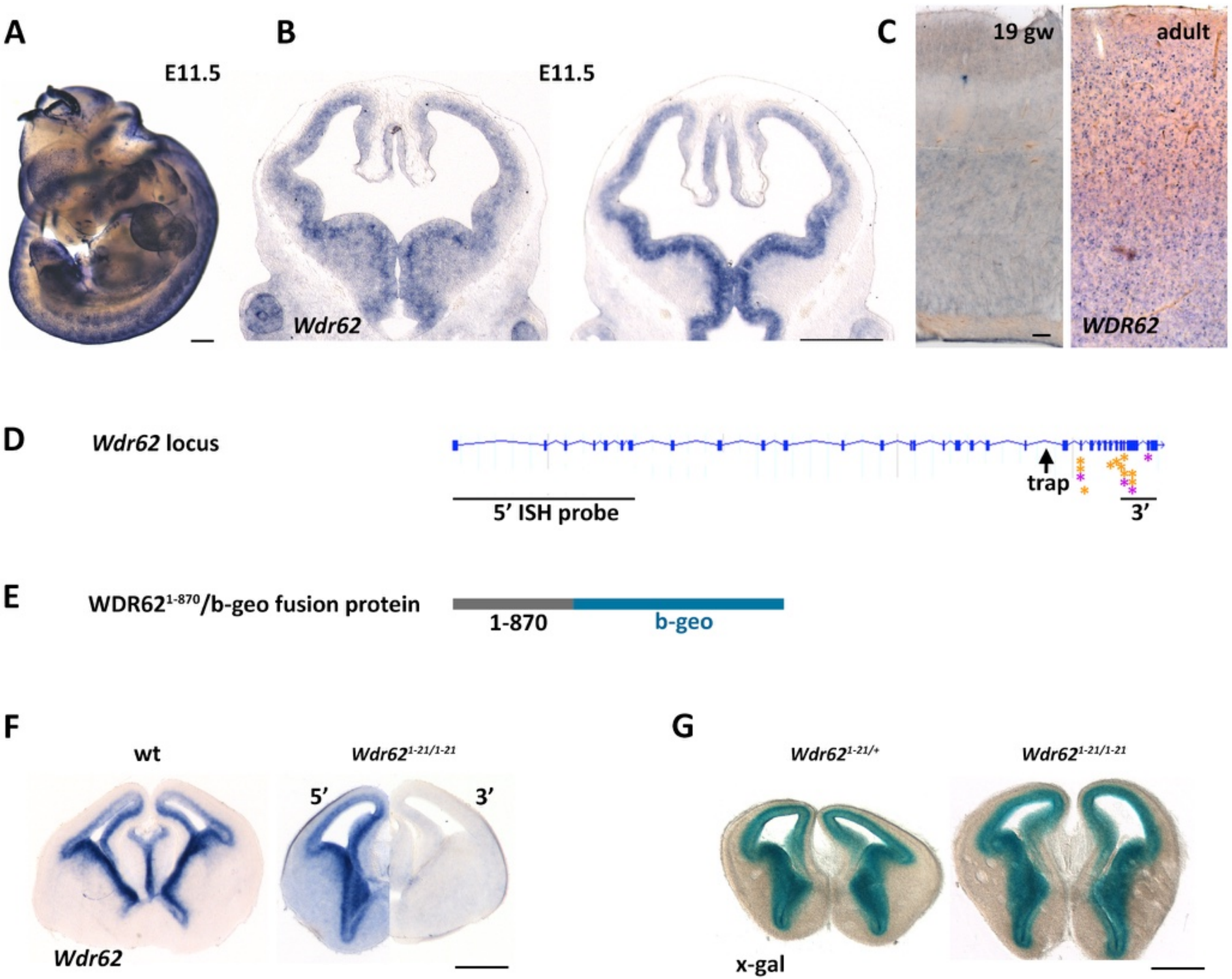

A
E11.5
B
E11.5
Wdr62
C
19 gw
adult
WDR62
D
Wdr62 locus
trap
5' ISH probe
3'
E
WDR62^1-870/b-geo fusion protein
1-870
b-geo
F
wt
Wdr62
Wdr62^1-21/1-21
5'
3'
G
Wdr62^1-21/+
Wdr62^1-21/1-21
x-gal

Supplementary Figure S1. **Generation of *Wdr62*$^{1\text{-}21/1\text{-}21}$ mice**

(A-C) Expression of *Wdr62* mRNA in murine embryonic and human fetal brain

(A) Representative image of a wild type embryo at E11.5 after whole mount in situ hybridization demonstrates *Wdr62* expression in the developing central nervous system and in non-neural tissues.

(B) Representative images of coronal sections at two different levels of the developing forebrain at E11.5 after in situ hybridization. *Wdr62* mRNA is highly expressed in neural progenitors in the VZ and SVZ in dorsal and ventral forebrain.

(C) Expression of *WDR62* in human brain detected by in situ hybridization at 19 weeks of gestation (gw) and adult temporal lobe (58 years). In fetal brain, *WDR62* mRNA is enriched in the VZ, and weakly present in the intermediate zone and, at low levels, in the cortical plate. In adult brain, *WDR62* is expressed throughout the cortical layers.

(D, E) Targeting of the *Wdr62* locus

(D) Genomic organization of *Wdr62.* Blue boxes represent exons. The insertion site of the pGTOlxr gene-trap vector (trap) between exons 21 and 22 is indicated with a black arrow. Asterisks indicate the locations of previously published (by us and others; orange stars) or newly identified (by us; purple stars) mutations affecting conserved amino acids in the human *WDR62* locus. Only mutations mapping downstream of the vector insertion site are shown. The locations of in situ hybridization probes, one at the 5' end of the transcript (5' ISH probe; exons 1-7), the other at the 3' end (3'; exons 29-33) are also indicated (D). Predicted structure of the fusion protein encoded by the targeted *Wdr62* allele. This trapped allele generates a carboxy-terminally truncated protein lacking the C-terminal 654 (of 1524) amino acids fused to β-geo.

(E) In situ hybridization with *Wdr62* antisense riboprobes on coronal sections of wild type (left) and *Wdr62*$^{1\text{-}21/1\text{-}21}$ developing forebrain from E14.5 littermates reveals normal expression of the fusion transcript (detected by the 5' ISH probe), but no expression of a full-length transcript (detected with the

3’ probe) in the latter. The hemisections of *Wdr62*$^{1\text{-}21/1\text{-}21}$ brain processed for in situ hybridization with the 5’ (left) and 3’ (right) probes are from the same embryo and shown juxtaposed for ease of comparison (F). X-gal staining of coronal sections of *Wdr62*$^{1\text{-}21/+}$ heterozygous and *Wdr62*$^{1\text{-}21/1\text{-}21}$ homozygous littermates at E13.5 demonstrates expression of the WDR62/ β-geo fusion protein (G).

Scale bar: (A, B): 0.5 mm; (C): 0.2 mm; (F, G): 0.5 mm

FIG. S2

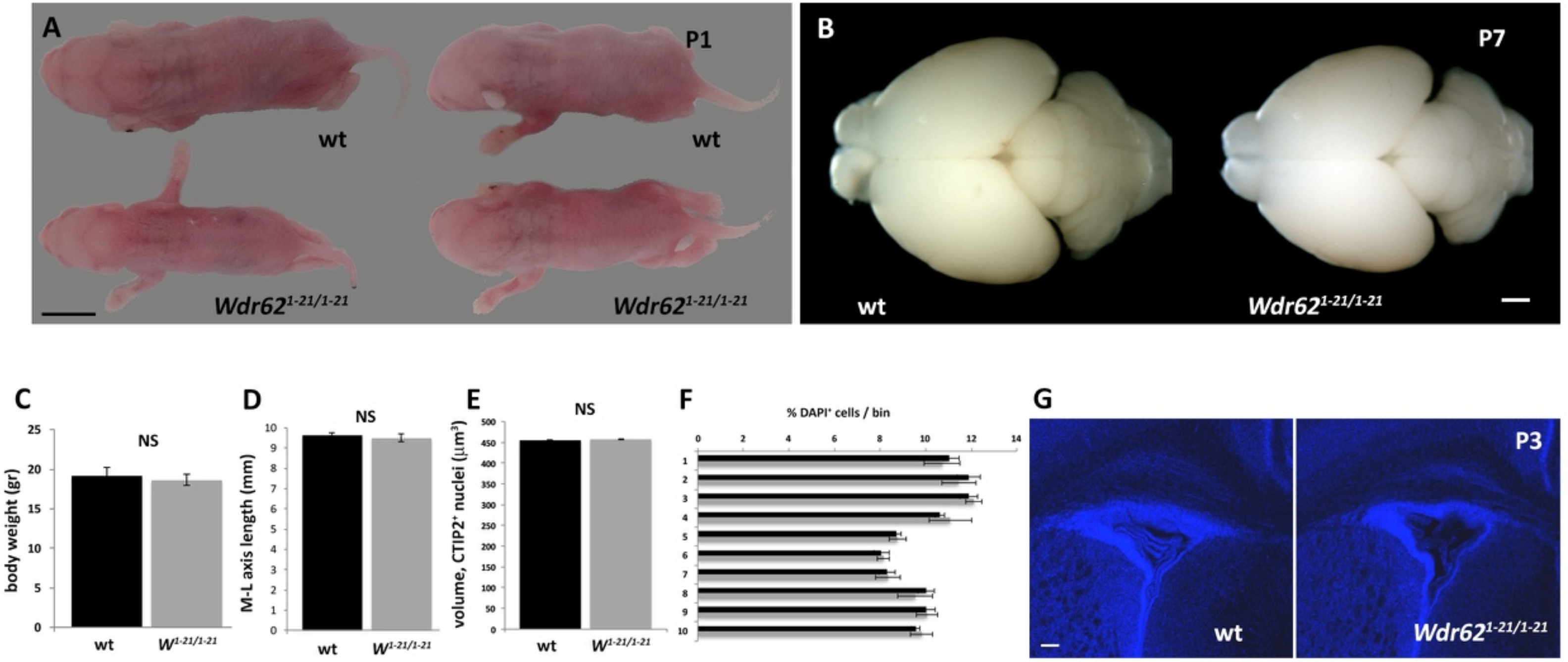

A
P1
wt
wt
Wdr62 1-21/1-21
Wdr62 1-21/1-21
B
P7
wt
Wdr62 1-21/1-21
C
NS
body weight (gr)
wt
W 1-21/1-21
D
NS
M-L axis length (mm)
wt
W 1-21/1-21
E
NS
volume, CTIP2+ nuclei (µm³)
wt
W 1-21/1-21
F
% DAPI+ cells / bin
G
P3
wt
Wdr62 1-21/1-21

Supplementary Figure S2. **Phenotypic characterization of *Wdr62*$^{1\text{-}21/1\text{-}21}$ homozygous mice**

(A, B) *Wdr62*$^{1\text{-}21/1\text{-}21}$ homozygous mice exhibit reduced body size and microcephaly at early postnatal stages

(A) Body size of *Wdr62*$^{1\text{-}21/1\text{-}21}$ pups is variably reduced compared with that of wild type littermates at P1. The two pairs of littermates shown represent extreme examples to illustrate the variability in size differences between wild type and *Wdr62*$^{1\text{-}21/1\text{-}21}$ neonates.

(B) Representative images of whole brains from wild type and *Wdr62*$^{1\text{-}21/1\text{-}21}$ littermates at P7.

(C, D) *Wdr62*$^{1\text{-}21/1\text{-}21}$ mice exhibit normal body weight and M-L axis length in adulthood (9 weeks)

(C) Measurements of body weight (n=4 pairs) show no differences between wild type and *Wdr62*$^{1\text{-}21/1\text{-}21}$ mice.

(D) No differences are detected in the M-L axis length (n= 4 pairs), despite significant differences in the A-P axis length (see Figure 1) between wild type and *Wdr62*$^{1\text{-}21/1\text{-}21}$ brains.

(E) The nuclear volume of CTIP2$^+$ cells at P3 (n= 4 pairs) is not significantly different in *Wdr62*$^{1\text{-}21/1\text{-}21}$ (456.64 ± 0.549) compared with wild type (455.27 ± 1.012) neocortex.

(F) Normal distribution of nuclei within the neocortical wall of wild type and *Wdr62*$^{1\text{-}21/1\text{-}21}$ littermates at P3, expressed as the fraction of total DAPI$^+$ cells residing within each of 10 equally sized bins (n= 3 pairs).

(G) The lateral ventricles at early postnatal stages (P3) mice often appear enlarged in *Wdr62*$^{1\text{-}21/1\text{-}21}$ compared with wild type littermates.

Scale bar: (A): 0.5 cm; (B): 1 mm; (F): 0.1 mm

Error bars represent s.e.m; NS: not significant（two-tailed Student's *t*-test)

FIG. S3

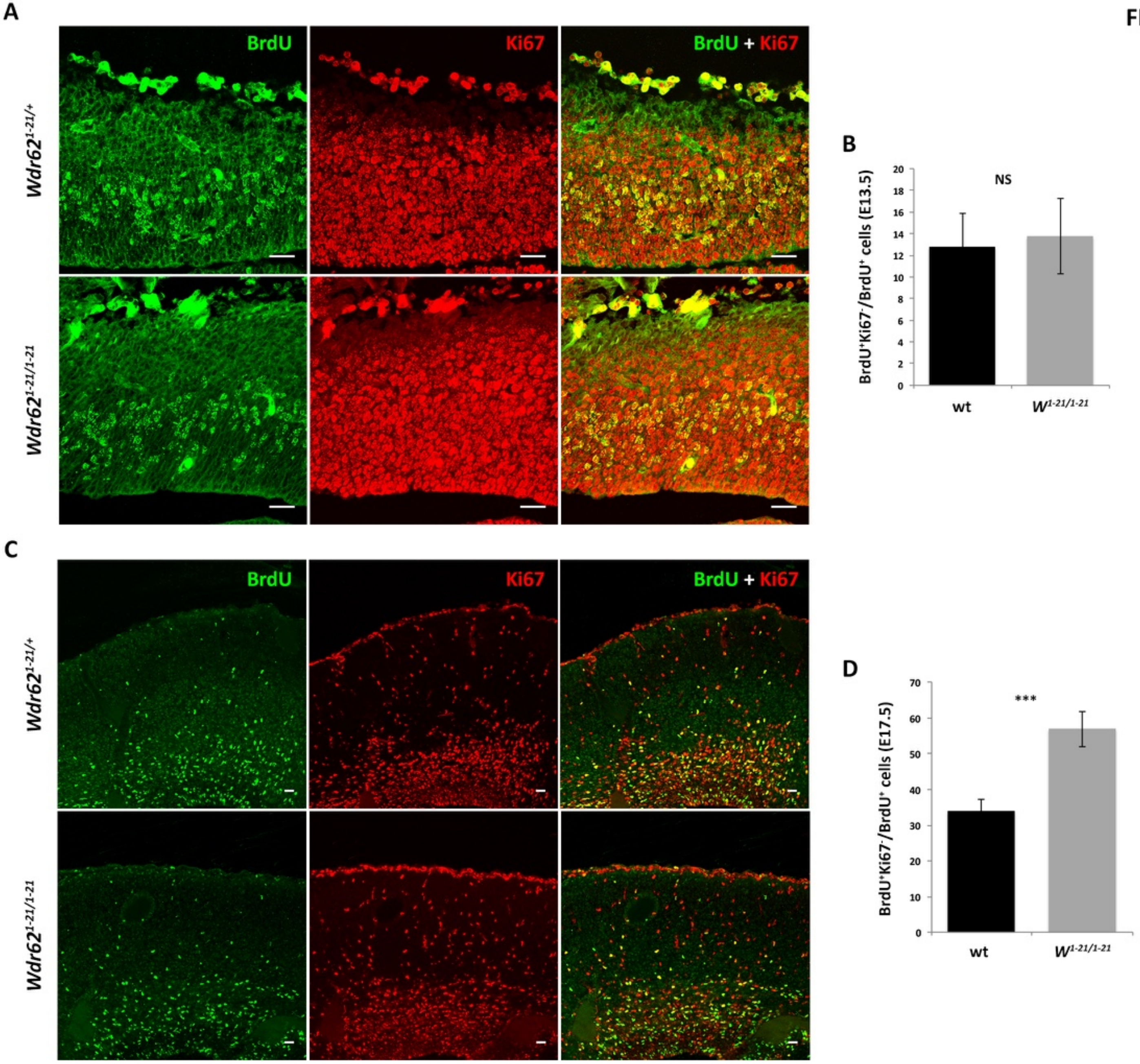
A
BrdU
Ki67
BrdU + Ki67
Wdr62 1-21/+
Wdr62 1-21/1-21
B
NS
BrdU+Ki67-/BrdU+ cells (E13.5)
20
18
16
14
12
10
8
6
4
2
0
wt
W1-21/1-21
C
BrdU
Ki67
BrdU + Ki67
Wdr62 1-21/+
Wdr62 1-21/1-21
D
***
BrdU+Ki67-/BrdU+ cells (E17.5)
70
60
50
40
30
20
10
0
wt
W1-21/1-21

Supplementary Figure S3. **Cell cycle abnormalities in *Wdr62*$^{1\text{-}21/1\text{-}21}$ neural progenitors**

(A-D) Analyses of BrdU incorporation (30-minute pulse).

(A, C) Coronal sections of heterozygous *Wdr62*$^{1\text{-}21/+}$ (A) and homozygous *Wdr62*$^{1\text{-}21/1\text{-}21}$ (C) embryonic neocortex at E13.5 (A) and E17.5 (C) immunostained with antibodies specific to BrdU and Ki67.

(B, D) Quantification of the number of BrdU^{+}Ki67^{-}/BrdU^{+} cells ("leaving fraction") at E13.5 (B) and E17.5 (D) reveals increased number of cells exiting the cell cycle during the 30-minute interval between BrdU administration and brain extraction in *Wdr62*$^{1\text{-}21/1\text{-}21}$ neocortex compared with heterozygous control at E17.5 (n=3, with 3-4 sections analyzed per brain).

Error bars represent s.e.m; ★★★ $p<0.005$; NS: not significant (two-tailed Student's *t*-test)

FIG. S4

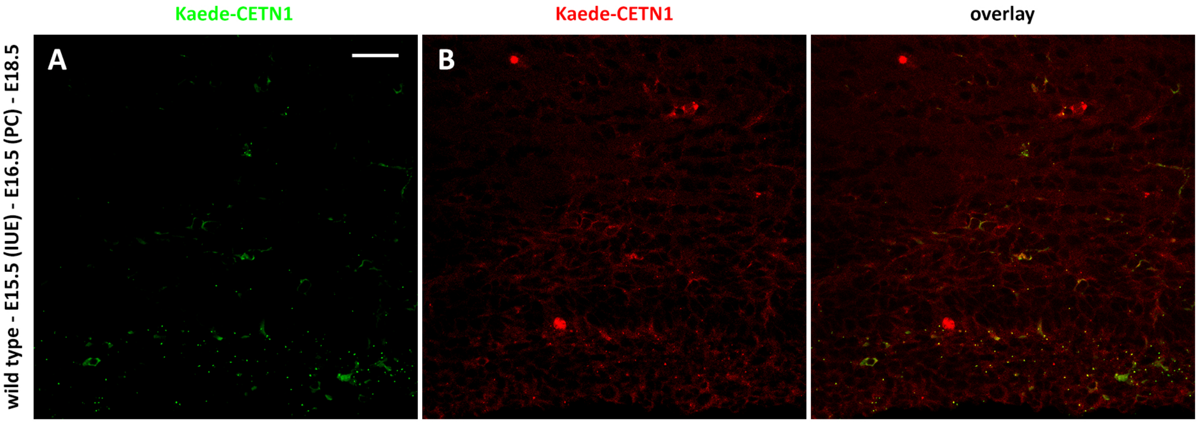
Kaede-CETN1
Kaede-CETN1
overlay
wild type - E15.5 (IUE) - E16.5 (PC) - E18.5
A
B

Supplementary Figure S4. **In vivo labeling of centrosomes using Kaede-CETN1**

(A-C) Representative, high magnification images of the VZ/SVZ (bin 1) area of the neocortical wall of E18.5 wild type embryos electroporated with Kaede-CETN1 at E15.5 and photoconverted at E16.5 (see Figure 3 for details). Images are acquired in the green (A) and red (B) channels, then overlaid (C). Each dot corresponds to a centrosome, which can be green-only, red-only or green-and-red (i.e. yellow)-fluorescent. The number of labeled centrosomes per color category is then quantified.

Scale bar (A, applies to all): 30 μm.

FIG. S5

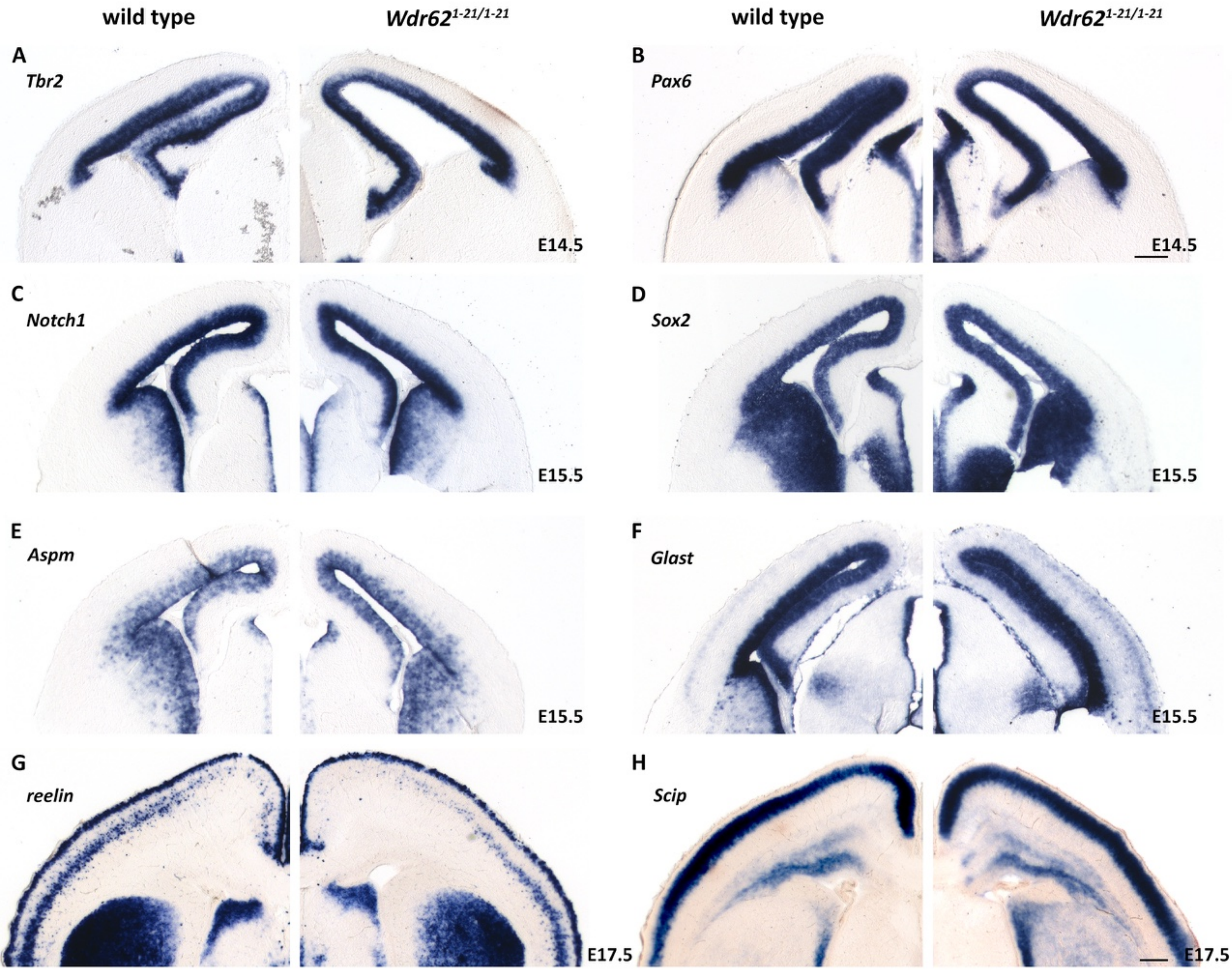
wild type
Wdr62^1-21/1-21
wild type
Wdr62^1-21/1-21
A
Tbr2
E14.5
B
Pax6
E14.5
C
Notch1
E15.5
D
Sox2
E15.5
E
Aspm
E15.5
F
Glast
E15.5
G
reelin
E17.5
H
Scip
E17.5

Supplementary Figure S5. **Expression analyses of neural progenitor and neuronal identity markers in embryonic neocortex**

(A-J) mRNA expression of molecular markers of neocortical progenitors and neurons.

Representative images of coronal sections of wild type and *Wdr62*$^{1\text{-}21/1\text{-}21}$ embryonic brain processed with in situ hybridization as indicated.

No obvious differences are observed in the expression of *Tbr2* (A), *Pax6* (B), *Notch1* (C), *Sox2* (D), *Aspm* (E), *Glast* (F), *reelin* (G) or *Scip* (H) between wild type and *Wdr62*$^{1\text{-}21/1\text{-}21}$ neocortex at E14.5 (A, B), E15.5 (C-F), or E17.5 (G, H).

Scale bar: B (also applies to A-F): 0.2 mm; H (also applies to C-H): 0.2 mm.

FIG. S6

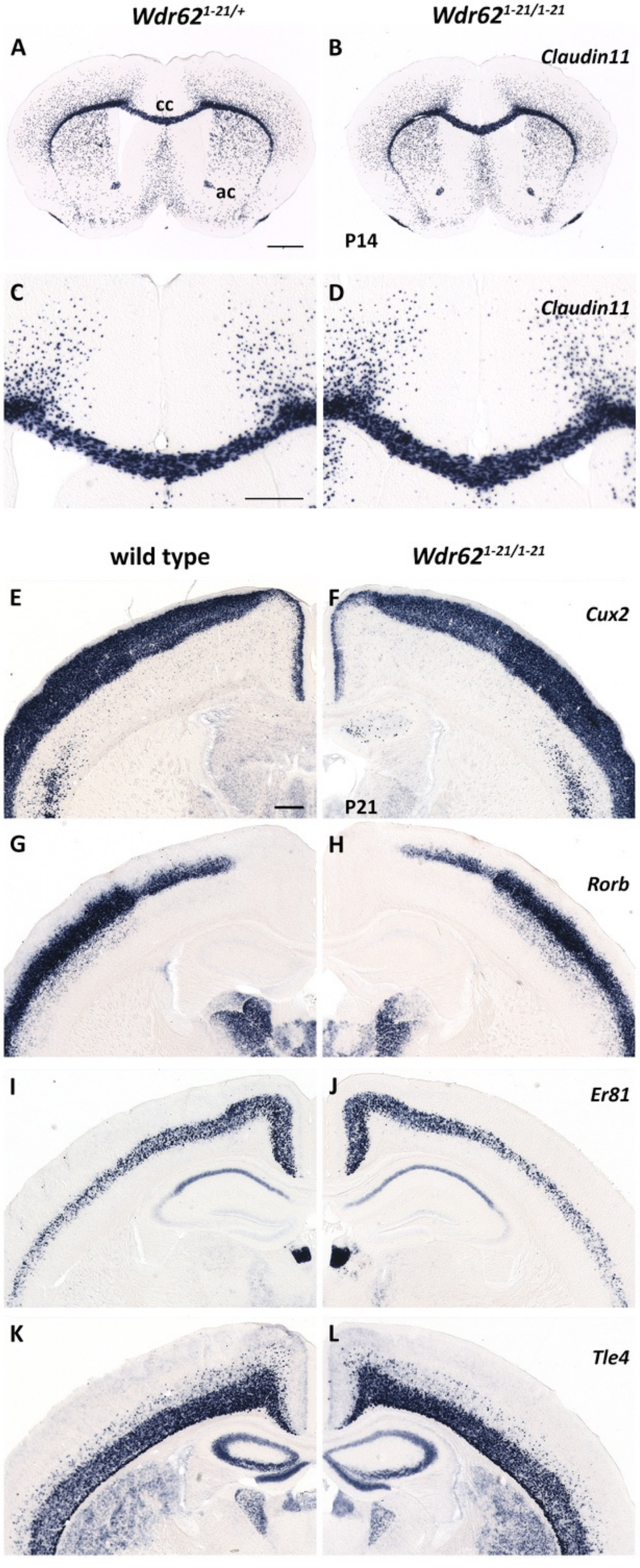
Wdr62^1-21/+
Wdr62^1-21/1-21
A
B
Claudin11
cc
ac
P14
C
D
Claudin11
wild type
Wdr62^1-21/1-21
E
F
Cux2
P21
G
H
Rorb
I
J
Er81
K
L
Tle4

Supplementary Figure S6. **Expression analyses in postnatal neocortex**

Representative images of coronal sections of wild type and *Wdr62*$^{1\text{-}21/1\text{-}21}$ brains processed with in situ hybridization as indicated.

(A-D) *Claudin11*, a marker of myelinating oligodendrocytes, and thus -indirectly- of axonal tracts, reveals that various commissures (e.g. the anterior commissure [ac] and the corpus callosum [cc], both shown here) form normally (A, B). There appears to be a slight increase in the thickness of the corpus callosum in *Wdr62*$^{1\text{-}21/1\text{-}21}$ brains (D) compared with wild type (C) at P14, in agreement with increased number of SATB2^{+} callosal neurons (see Figure 4).

(E-L) mRNA expression of cortical layer specific markers, *Cux2* (E, F), *Rorb* (G, H), *Er81* (I, J) and *Tle4* (K, L) is grossly normal in *Wdr62*$^{1\text{-}21/1\text{-}21}$ (F, H, J, L) neocortex compared with wild type (E, G, I, K) at P21.

Scale bar: A (also applies to B): 0.2 mm; C (also applies to D): 0.5 mm; E (also applies to F-L): 0.5 mm.

FIG. S7

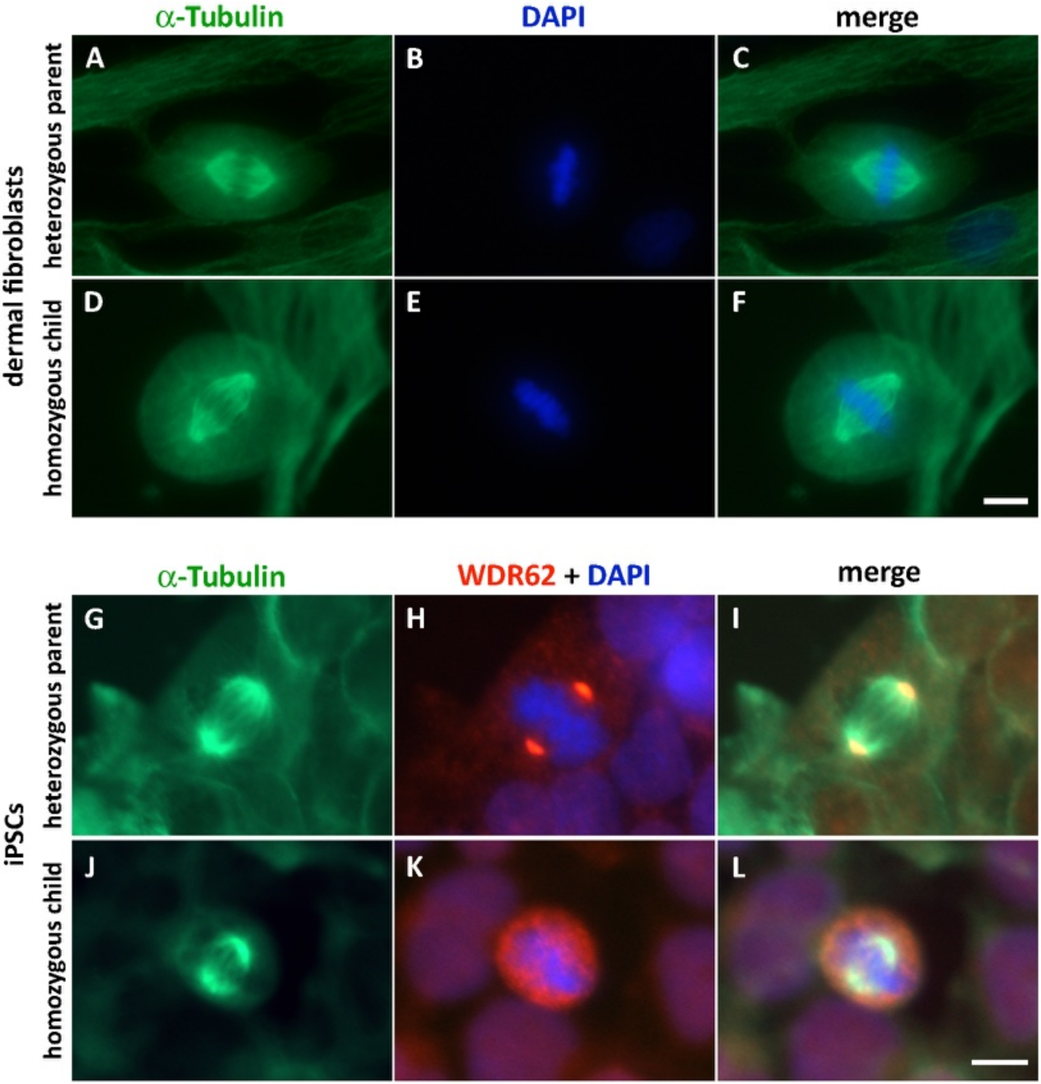

α-Tubulin
DAPI
merge
dermal fibroblasts
heterozygous parent
homozygous child
A
B
C
D
E
F
α-Tubulin
WDR62 + DAPI
merge
iPSCs
heterozygous parent
homozygous child
G
H
I
J
K
L

Supplementary Figure S7. **Morphology of the mitotic spindle in dermal fibroblasts and iPSCs from family NG1406 carrying the *WDR62*^{exon23} missense mutation**

Dermal fibroblasts were established from skin biopsies obtained from the heterozygous parents and two affected (homozygous) children from family NG1406, carrying a missense mutation (D955AfsX112) in exon 23 of *WDR62.*

(A-F) Immunofluorescent staining of dermal fibroblasts undergoing mitosis in unsynchronized cultures for α-tubulin. Spindle morphology is normal in *WDR62*^{exon23} heterozygous (A-C) and homozygous (D-F) fibroblasts.

(G-L) Immunofluorescent staining of iPSCs undergoing mitosis for α-tubulin and WDR62. Spindle morphology is normal in heterozygous (G-I) and *WDR62*^{exon23} homozygous (J-L) iPSCs. WDR62 localizes to the spindle poles in heterozygous cells but remains diffusely distributed in the cytoplasm in homozygous cells.

Scale bars (F, applies to A-E): 20 μm; (L, applies to G-K): 20 μm.

FIG. S8

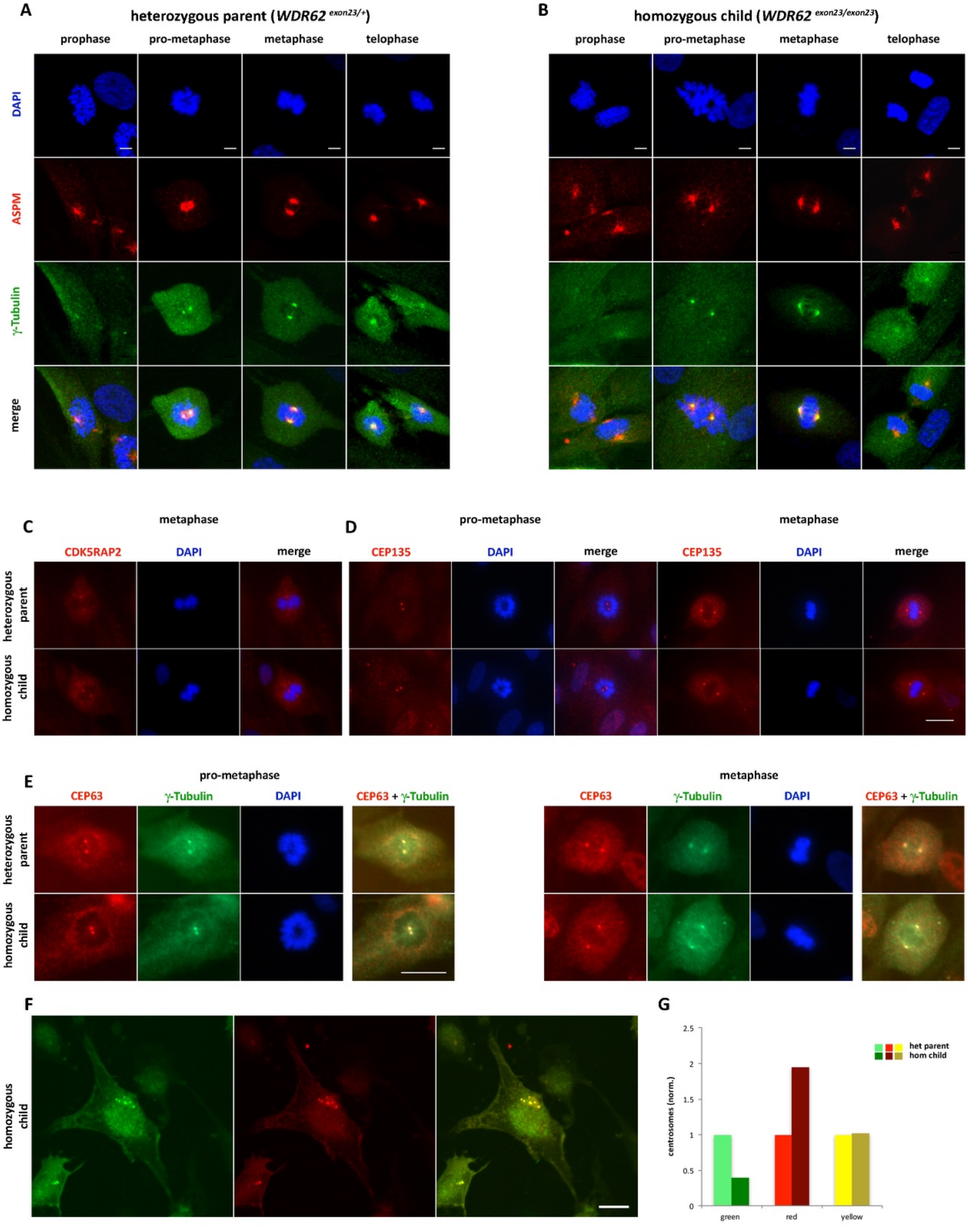
A
heterozygous parent (WDR62 exon23/+)
prophase
pro-metaphase
metaphase
telophase
DAPI
ASPM
γ-Tubulin
merge
B
homozygous child (WDR62 exon23/exon23)
C
metaphase
CDK5RAP2
heterozygous parent
homozygous child
D
pro-metaphase
CEP135
E
CEP63
CEP63 + γ-Tubulin
F
G
centrosomes (norm.)
het parent
hom child
green
red
yellow
0
0.5
1
1.5
2
2.5

Supplementary Figure S8. **Characterization of dermal fibroblasts from family NG1406**

(A, B) Immunofluorescent staining of dermal fibroblasts undergoing mitosis in unsynchronized cultures for ASPM, a spindle pole protein, and γ-tubulin. ASPM localization to the spindle poles, where it overlaps with γ-tubulin, is not altered in *WDR62*^{exon23} homozygous (B) as compared with heterozygous (A) fibroblasts at any of the mitotic phases (representative images as indicated).

(C, D) Immunofluorescent staining of dermal fibroblasts with the pericentriolar protein CDK5RAP2 (C) and the centrosomal protein CEP135 (D). Expression and localization of both markers are similar in *WDR62*^{exon23} heterozygous (top row) and homozygous (bottom row) fibroblasts during mitosis (only select representative images of cells in metaphase and/or pro-metaphase are shown, as indicated).

(E) Immunofluorescent staining of dermal fibroblasts with the centrosomal protein CEP63 and γ-tubulin. Expression and localization of CEP63 is similar in *WDR62*^{exon23} heterozygous (top row) and homozygous (bottom row) fibroblasts during mitosis (only select, representative images or cells in prometaphase and metaphase are shown, as indicated).

(F) Rare example of a *WDR62*^{exon23} homozygous fibroblast cell harboring multiple centrosomes. Fibroblasts were transfected with Kaede-CETN1 to pulse-label centrosomes and photoconverted 24 hours post-nucleofection (see Methods). The cell displayed contains multiple centrosomes consisting of green- and red-fluorescent centrioles.

(G) Normalized number of centrosomes that are green-only (containing new mother centrioles only), red-only (having not divided) or yellow (containing one old [red] and one new [green] mother centrioles indicates that comparatively more centrosomes have not divided in *WDR62* homozygous vs. heterozygous fibroblasts. (n= 401 and n= 411 centrosomes were counted, respectively, in heterozygous vs. homozygous cells).

Scale bar: (A, B; applies to all panels): 5 μm; (C, D; applies to all panels): 0.02 mm; (E; applies to all panels): 0.02 mm; (F): 20 μm.

FIG. S9

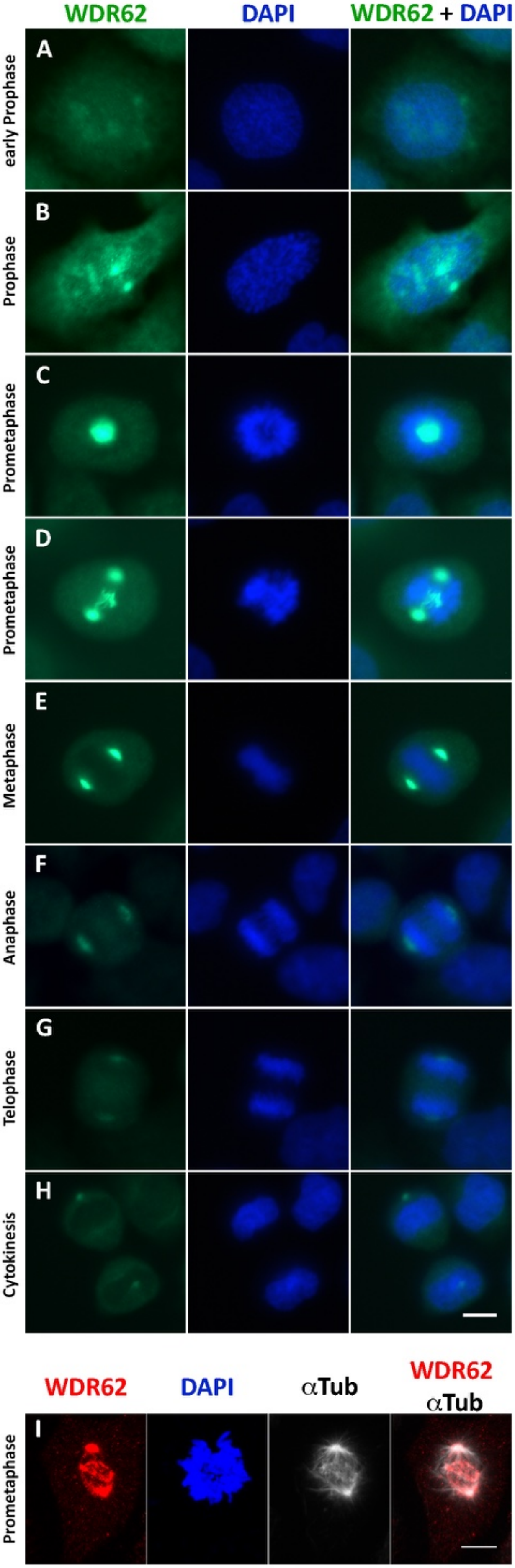
WDR62
DAPI
WDR62 + DAPI
A
early Prophase
B
Prophase
C
Prometaphase
D
Prometaphase
E
Metaphase
F
Anaphase
G
Telophase
H
Cytokinesis
WDR62
DAPI
αTub
WDR62
αTub
I
Prometaphase

Supplementary Figure S9. **Characterization of WDR62 expression in HeLa cells during mitosis**

(A-H) Immunofluorescence staining of HeLa cells undergoing mitosis with an antibody specific to WDR62. Representative images of cells at all phases of the mitotic cycle (as indicated). WDR62 is more prominent in HeLa cells compared to dermal fibroblasts, with expression foci appearing during early prophase, progressively intensifying in prometaphase, a stage at which WDR62 is still dynamically distributed without being exclusively associated with the spindles poles. From metaphase onward, WDR62 becomes primarily localized to the spindle poles, where it persists at low levels until cytokinesis.

(I) Immunofluorescence staining of HeLa cells at prometaphase with antibodies specific to WDR62 and a Tubulin indicates WDR62 association with the spindle.

Scale bar: (H, applies to A-H): 20 μm; (I): 10 μm.

FIG. S10

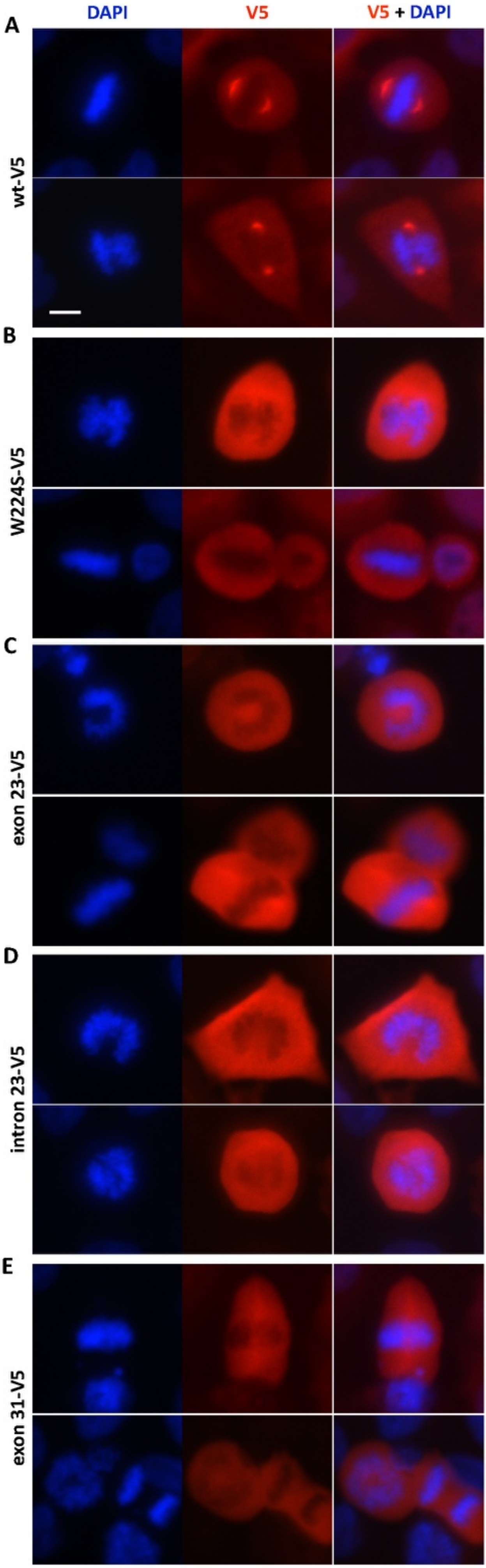

DAPI
V5
V5 + DAPI
A
wt-V5
B
W224S-V5
C
exon 23-V5
D
intron 23-V5
E
exon 31-V5

Supplementary Figure S10. **MCPH-associated mutations disrupt the subcellular localization of WDR62 during mitosis in HeLa cells**

(A-E) Immunofluorescence staining of HeLa cells transiently transfected with expression plasmids encoding wild type and mutant forms of human WDR62 labeled with a carboxyl-terminal V5 tag (Methods). Additional details on the MCPH-associated mutant forms of WDR62 analyzed are provided in Methods. Upon overexpression, wild type WDR62 (wt-V5, A), detected with an antibody specific to V5, recapitulates the expression and localization pattern of endogenous WDR62 at the spindle poles during prometaphase and metaphase (other mitotic phases not shown). In contrast, none of the four mutant forms of WDR62 examined, also visualized with V5 (W224S-V5 [B]; D955AfsX112 - "exon 23-V5" [C]; S956CfsX38 - "intron 23-V5" [D]; V1402GfsX12 - "exon 31-V5" [E]) is detected in the spindle poles at any of the mitotic phases, as evidenced by diffuse cytoplasmic staining.

Scale bar: (A, applies to all panels): 20 μm.

FIG. S11

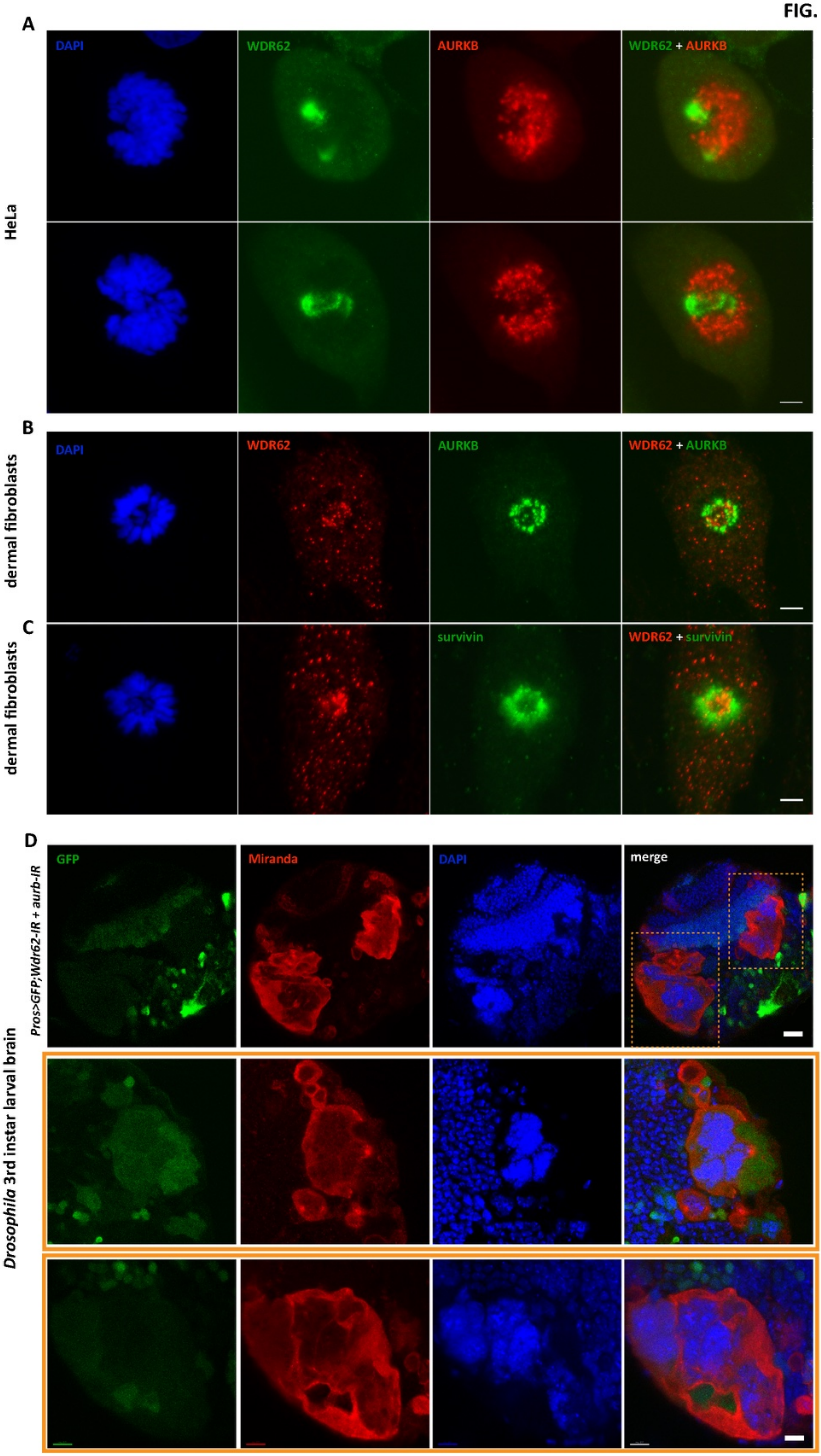
A
HeLa
DAPI
WDR62
AURKB
WDR62 + AURKB
B
dermal fibroblasts
DAPI
WDR62
AURKB
WDR62 + AURKB
C
dermal fibroblasts
survivin
WDR62 + survivin
D
Pros>GFP;Wdr62-IR + aurb-IR
Drosophila 3rd instar larval brain
GFP
Miranda
DAPI
merge

Supplementary Figure S11.

(A-C) Characterization of WDR62, AURKB and survivin expression during mitosis in HeLa cells and dermal fibroblasts

(A) Immunofluorescence staining of HeLa cells undergoing mitosis with antibodies specific to WDR62 and AURKB. Representative images of cells at prometaphase demonstrate partial and transient co-localization of the two proteins.

(B, C) Immunofluorescence staining of dermal fibroblasts undergoing mitosis with antibodies specific to WDR62 and AURKB (B) or survivin (C). Representative images of cells at prometaphase demonstrate partial and transient co-localization of WDR62 with each of the two CPC core proteins.

(D) Enlarged neuroblasts following combined *wdr62-IR* and *aurb-IR* in *Drosophila* 3rd instar larval brain. A single section through the combined knockdown (*Wdr62-IR* and *Aurb-IR*) 3rd instar larval brain (Fig. 6L) is shown in the top row. Higher magnifications of the abnormally enlarged neuroblasts (dashed boxes) are shown in the bottom two rows. Neuroblasts are stained with miranda (red) and nuclei with DAPI (blue).

Scale bar (A-C): 5 μm; (D): 200 μm (top row), 5 μm (bottom rows).

Supplementary Table S1. Transcripts with circadian expression highly correlated to *WDR62*

Gene Symbol	RefSeq	Gene Name	Function
**ESPL1**	NM_012291	Extra spindle poles-like 1	Separase
**IQGAP3**	NM_178229	IQ motif containing GTPase activating protein 3	
**SHCBP1**	NM_024745	Shc SH2-domain binding protein 1	Cell proliferation
**KIF2C**	NM_006845	Kinesin family member 2C	CPC substrate
**CDCA2**	NM_152562	Cell division cycle associated 2	Mitosis
**MELK**	NM_014791	Maternal embryonic leucine zipper kinase	Cell cycle
**CDCA8**	NM_018101	Cell division cycle associated 8	CPC core
**BIRC5**	NM_001168	Baculoviral IAP repeat-containing 5	CPC core
**TACC3**	NM_006342	Transforming acidic coiled-coil containing kinase 3	Mitosis
**NCAPH**	NM_015341	Non-SMC condensing I complex, subunit H	Mitosis
**AURKB**	NM_004217	Aurora kinase B	CPC core
**NEK2**	NM_002497	NIMA-related expressed protein 2	Mitosis
**KIF20A**	NM_005733	Kinesin family member 20A	Cytokinesis
**HMMR**	NM_001142556	Hyaluroan mediated motility receptor	Cell motility
**CENPE**	NM_001813	Centromere protein E	CPC substrate
**TTK**	NM_003318	TTK protein kinase	CPC interactor
**PBK**	NM_018492	PDZ binding kinase	
